# Field multi-omics analysis reveals a close association between bacterial communities and mineral properties in the soybean rhizosphere

**DOI:** 10.1038/s41598-021-87384-8

**Published:** 2021-04-23

**Authors:** Shinichi Yamazaki, Hossein Mardani-korrani, Rumi Kaida, Kumiko Ochiai, Masaru Kobayashi, Atsushi J. Nagano, Yoshiharu Fujii, Akifumi Sugiyama, Yuichi Aoki

**Affiliations:** 1 Tohoku Medical Megabank Organization, Tohoku University, Sendai, Japan; 2 Department of International Environmental and Agricultural Science, Tokyo University of Agriculture and Technology, Fuchu, Japan; 3 Division of Applied Life Sciences, Graduate School of Agriculture, Kyoto University, Kyoto, Japan; 4 Faculty of Agriculture, Ryukoku University, Otsu, Japan; 5 Research Institute for Sustainable Humanosphere, Kyoto University, Gokasho, Uji, Japan

**Keywords:** Plant sciences, Environmental microbiology

## Abstract

The plant root-associated environments such as the rhizosphere, rhizoplane, and endosphere are different from the outer soil region (bulk soil). They establish characteristic conditions including microbiota, metabolites, and minerals, and they can directly affect plant growth and development. However, comprehensive insights into those characteristic environments, especially the rhizosphere, and molecular mechanisms of their formation are not well understood. In the present study, we investigated the spatiotemporal dynamics of the root-associated environment in actual field conditions by multi-omics analyses (mineral, microbiome, and transcriptome) of soybean plants. Mineral and microbiome analyses demonstrated a characteristic rhizosphere environment in which most of the minerals were highly accumulated and bacterial communities were distinct from those in the bulk soil. Mantel’s test and co-abundance network analysis revealed that characteristic community structures and dominant bacterial taxa in the rhizosphere significantly interact with mineral contents in the rhizosphere, but not in the bulk soil. Our field multi-omics analysis suggests a rhizosphere-specific close association between the microbiota and mineral environment.

## Introduction

Plants cannot autonomously move from their planting place, so they must take up almost all essential nutrients for growth and development via their roots from the soil. Most mineral nutrients contained in the soil, however, are not in an accessible or available form to plants. For instance, 90% of soil nitrogen in a forest ecosystem is contained in organic matter^1^ such as proteins and humus^2,3^, which cannot be utilized by plants as they are. Soil microbes therefore play essential roles in the chemical transformation of the unavailable nitrogen into its available, inorganic form (mineralization)^4^, and they also facilitate solubilization of other nutrients including phosphate, potassium, and iron^5–7^. Plants themselves can solubilize soil minerals by exudation of organic acid^8^ and can communicate with soil microbes by secretion of organic compounds^9^. This means that plants interact with soil via microbes and metabolites.

The plant root-associated environments such as the rhizosphere, rhizoplane, and endosphere are quite different from the outer soil region known as bulk soil. Of these, the rhizosphere, an interface region between plant roots and bulk soil, is defined as the soil that is directly influenced by roots^10,11^. Many studies describe its characteristic environment in terms of microbiota, metabolites, and minerals. Previous studies show that plants release significant amounts of organic matter, accounting for more than 10% of photosynthetic products, from their roots into the soil^12^. Such richness of carbon in the rhizosphere stimulates the propagation of soil microbes, making the rhizosphere a microbial hotspot as one of the most dynamic habitats on earth^13,14^. Plant roots also secrete a variety of secondary metabolites including bioactive flavonoids into the rhizosphere. In legumes, flavonoids play a central role in communication with symbiotic nitrogen-fixing rhizobia^15^ and act as antimicrobial substances, phytoalexins, to inhibit root pathogens^16,17^. A recent study also revealed that an isoflavone, daidzein, secreted from soybean (*Glycine max*) roots was involved in shaping the rhizosphere bacterial community^18^. Thus, as shown in many studies, the microbial community structure of the rhizosphere is distinct from that in the bulk soil. In addition, under identical soil conditions, different plant species and cultivars shape different community structures in the rhizosphere^19,20^, suggesting that plants actively modify rhizosphere microbial communities in a genotype-specific manner, probably through secretion of metabolites^21^. Water and mineral concentrations are also influenced by plant uptake in the rhizosphere. Previous studies showed that some nutrient concentrations such as nitrogen, phosphorus, and potassium exhibit decreasing gradients between the bulk soil and the root surface^22,23^. By contrast, some excess elements such as calcium, magnesium, and iron accumulate around the root if their uptake is lower than the supply from mass flow^24–26^. In addition, the availability of minerals in the rhizosphere is also affected by microbial activity. These characteristic biological and chemical environments in the root-associated compartments can directly affect the plant growth and may reflect the physiological state of the host plant. Therefore, a comprehensive understanding of those environments is important for healthy plant growth and sustainable crop production, especially under environmental changes.

However, the root-associated environments have been independently characterized by their respective aspects of minerals, microbiota, and metabolites. The extent of the effect is variable depending on each biological and chemical factor and environmental condition. Furthermore, each factor is interacted with and affected by host plant activity. Such variable interactions between each component make it more complex; thus, holistic insight into the root-associated environments and molecular mechanisms of their formation need to be understood by the integration of diverse omics data. For an instance, a recent report demonstrates that multi-comics analysis can find key component to increase crop production on an agroecosystem^27^. It also remains unclear how a change in the rhizosphere caused by environmental perturbation affects plant growth and crop production.

In the present study, we performed a field cultivation experiment of soybeans with an application of cover crops, hairy vetch (*Vicia villosa*), as an environmental perturbation factor. Hairy vetch is a leguminous plant agriculturally used to improve soil fertility^28,29^ and produces allelochemicals to affect the surrounding environment. To gain a comprehensive insight into the root-associated environments, especially the interaction between biological and chemical components, in an actual field condition, we performed a time-series analysis of mineral composition in the rhizosphere, root-associated microbiome, and transcriptome of soybean plants. Cross-sectional analysis of those multi-omics data and analysis of spatiotemporal dynamics of respective factors revealed a significant interaction between the characteristic bacterial community and the mineral environment in the rhizosphere.

## Results

### Hairy vetch applications had a limited beneficial effect on soybean growth

**Figure 1:**
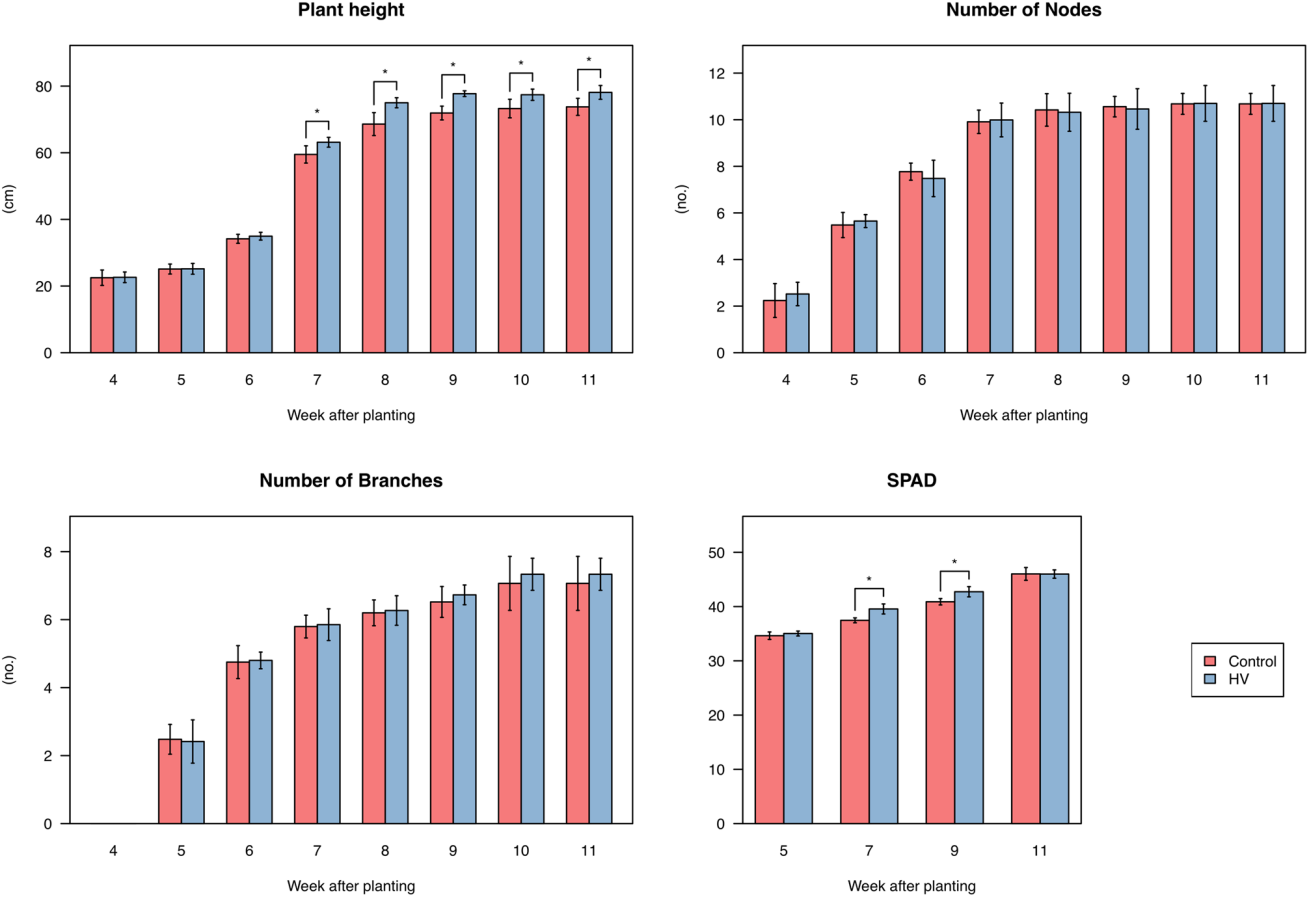
Growth indexes of soybean plants. Values are expressed as means ± SD (n = 5). Asterisks (*) show significant differences between the control plots and HV plots (*p* < 0.05, *t*-test). HV, hairy vetch; SPAD, soil plant analysis development.

We began a cultivation experiment of soybeans in a field at the beginning of June, and we regularly measured growth parameters such as plant height, number of nodes and branches, and soil plant analysis development (SPAD) from 4 to 11 weeks after planting (WAP; Fig. 1). The dry weights of shoots and roots were also measured after sampling of the rhizosphere (Supplementary Fig. S1). Soybean growth was slightly enhanced by the application of hairy vetch (HV). Plant height with HV application was slightly higher than that of the control plants at middle and later growth stages, and SPAD values with HV were also higher than those of the control (Fig. 1). However, the difference was limited, and other growth indexes including the dry weight of plants were not significantly different (Fig. 1, Supplementary Fig. S1).

### Mineral contents in the soybean rhizosphere and bulk soil

**Figure 2:**
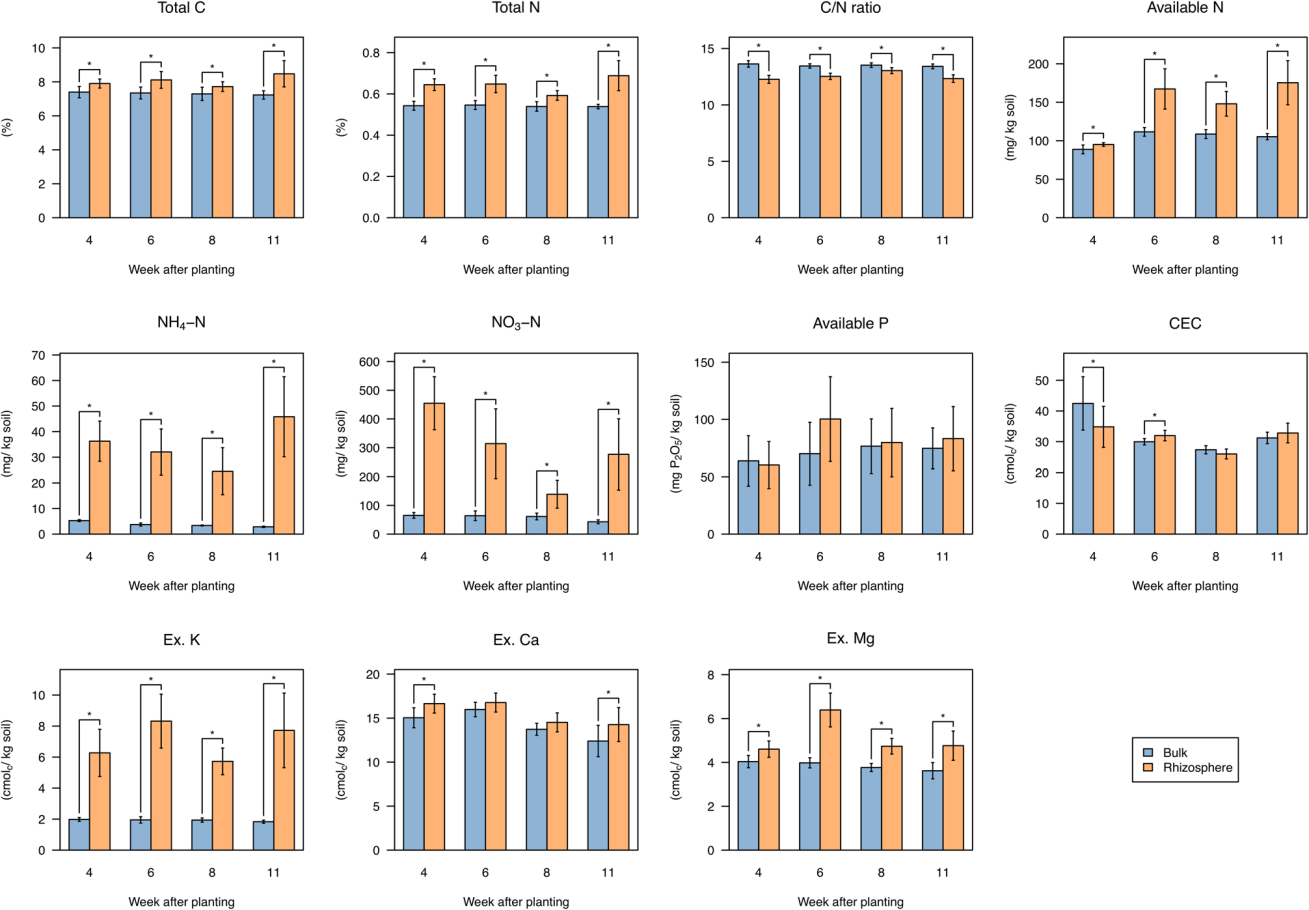
Mineral contents in bulk soil and rhizosphere soil of field-grown soybean plants. Values are expressed as means ± SD (n = 10). Asterisks (*) show significant differences between the bulk and rhizosphere soil (*p* < 0.05, *t*-test). CEC, cation exchange capacity; Ex, exchangeable.

We then analyzed the concentrations of mineral nutrients in the rhizosphere and bulk soil at 4, 6, 8, and 11 WAP, corresponding with vegetative (V)2, reproductive (R)1, R2, and R5 stages of soybean development, respectively. Using a small-scale protocol of mineral analysis for rhizosphere soil^30^, we measured the composition of principal macronutrients for plants: total C, total N, C/N ratio, available N, inorganic N (NH_4_-N and NO_3_-N), available P, cation exchange capacity (CEC), and exchangeable bases (K, Ca, and Mg). Most of the mineral contents in the rhizosphere soil were more abundant than those in the bulk soil; inorganic N and exchangeable K especially accumulated at high levels in the rhizosphere soils (Fig. 2). By contrast, available P was not significantly different between the soil regions at any of the time point tested (Fig. 2). Principal component analysis (PCA) of mineral contents showed that bulk soil and rhizosphere soil were discriminated by the first principal component (PC1; Supplementary Fig. S2a), and exchangeable K, total N, and NH_4_-N were the factors with large loading in PC1 (0.969, 0.956, and 0.943, respectively; Supplementary Fig. S3).

The NO_3_-N content in the HV-treated bulk soil was significantly higher than that of the control bulk soil, and NO_3_-N in the rhizosphere soils also exhibited a similar tendency between treatment groups (Supplementary Figs. S4, S5). Besides, CEC in the bulk soil was significantly different between treatments, but consistent trend was not observed during cultivation (Supplementary Fig. S4). No other mineral contents were significantly affected by HV application.

### Transcriptome analysis of the soybean leaf and root

We performed RNA-seq analysis of the leaves and roots of soybean plants collected on the same day of the rhizosphere sampling. Of the 56,044 genes in the Glyma v2.0 (Wm82.a2.v1) genome annotation, a total of 49,630 transcripts were detected in at least one sample. PCA for those data demonstrated that the gene expression profiles were clearly distinguished between the tissues and among plant growth stages (Supplementary Fig. S2b). Notably, one sample of the root at 4 WAP was distinct from the other root samples in the PCA plot (Supplementary Fig. S2b), probably because its quality of reads was very low; thus, we removed this root sample from subsequent analysis.

Differentially expressed genes (DEGs) under HV treatment were examined, and 337 DEGs were found in the leaf at 11 WAP, of which 165 genes were upregulated and 172 genes were downregulated under HV treatment (Additional file 1). We performed gene ontology (GO) enrichment analysis for these DEGs, but reasonable biological insight into the effect of HV treatment was not identified. In the other growth stages, only a few DEGs were detected. Thus, the effect of HV application on plant physiological status was limited.

**Figure 3:**
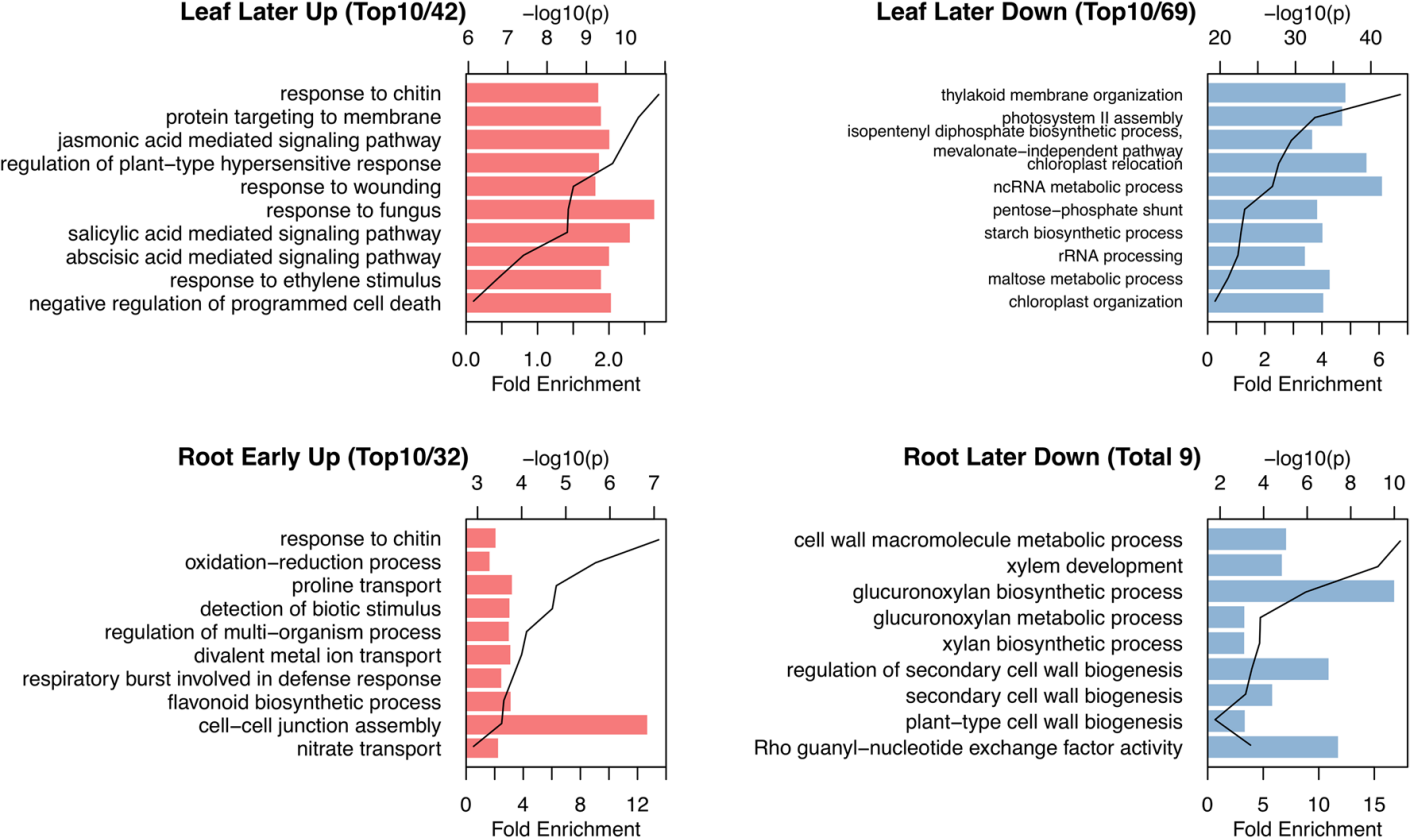
Gene ontology enrichment analysis of differential expression genes at early or later growth stage in the leaf and root. Gene expression profiles were classified into six patterns; significant increase or decrease (FDR < 0.05; fold change ≥ 2) only between 4 and 6 WAP (early stage), 6 and 8 WAP (mid stage), or 8 and 11 WAP (later stage) and no significant difference at the other stage. GO enrichment analysis was carried out using the Soybase GO term enrichment tool (https://www.soybase.org). GO, gene ontology; FDR, false discovery rate; WAP, weeks after planting.

To examine gene expression profiles during the development of soybean plants in field conditions, we compared the DEGs between different growth stages in our field data with previously reported laboratory-scale data^31^. The previous study characterized gene expression patterns by GO enrichment analysis among leaf developmental stages of soybean plants grown in pots in a growth chamber from V3 stage to the reproductive stage^31^, which corresponded to our sampling schedule in the field. The whole data of the GO enrichment analysis are shown in Additional file 2 and parts of these are listed in Fig. 3. In our field data, the genes associated with photosynthesis were downregulated, and those associated with responses to chitin and fungus were upregulated in the leaves at a later reproductive stage between 8 and 11 WAP (Fig. 3); these findings were consistent with the laboratory-scale data^31^. By contrast, expression patterns specific to the field were also observed. The genes associated with the signaling pathway mediated by jasmonic acid, salicylic acid, and abscisic acid were upregulated in the leaves between 8 and 11 WAP (Fig. 3), whereas these were not detected in the previous laboratory study. Gene expression patterns in the roots were quite different from those in the leaves. The GO terms of “divalent metal ion transport,” “nitrate transport,” and “cellular cation homeostasis” were enriched in the roots at the vegetative stage between 4 and 6 WAP (Fig. 3), indicating that the young seedlings actively took up mineral nutrients. At the later reproductive stage between 8 and 11 WAP, the genes associated with the cell wall biosynthetic process were significantly downregulated in the roots.

### Microbiome analysis of the root-associated bacteria

**Figure 4:**
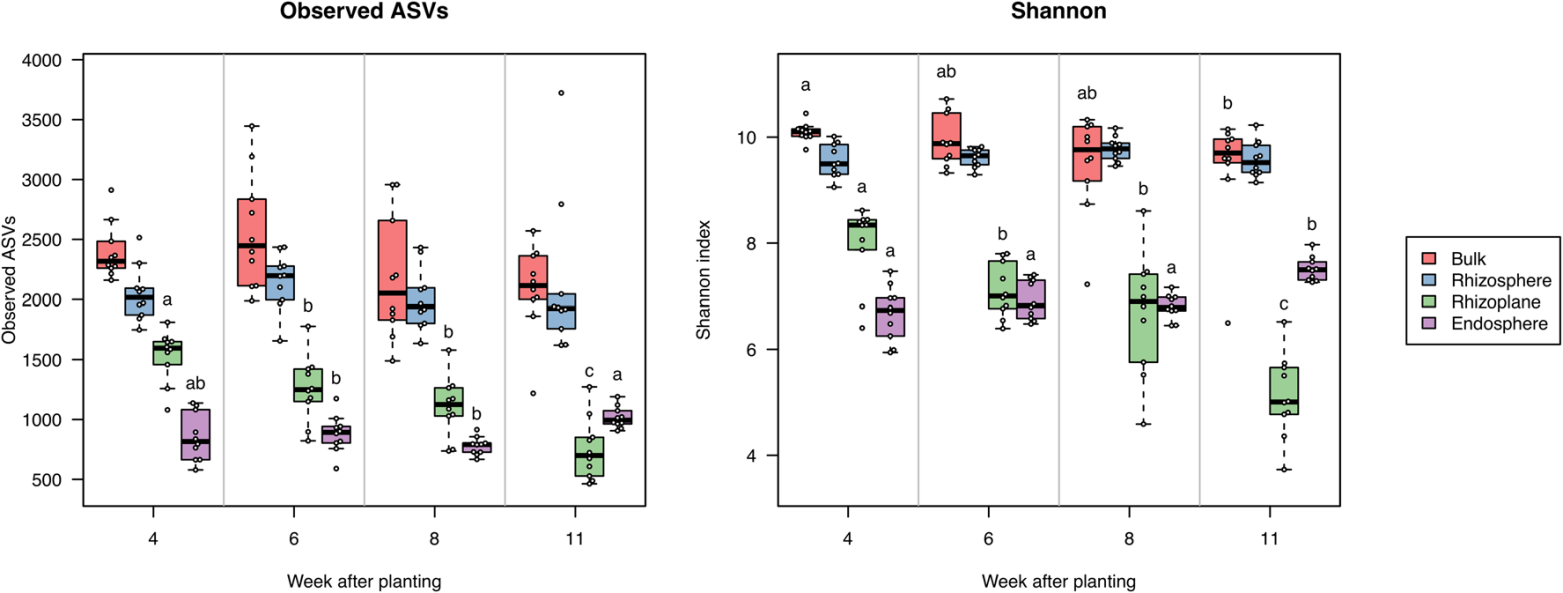
Alpha diversity of root-associated bacterial communities at different growth stages of field-grown soybean plants. Boxes showing different letters above them are significantly different among different growth stages (*p* < 0.05, Kruskal–Wallis test with Benjamini–Hochberg method).

To examine the profile of root-associated microbiota during plant development in the field, we analyzed the bacterial communities at the four root-associated compartments: bulk soil, rhizosphere, rhizoplane, and endosphere. After processing sequences by QIIME2 and removing chloroplastic and mitochondrial sequences, a total of 16,133,383 reads were obtained with the range of 52,780–321,718 reads per sample, representing 28,370 amplicon sequence variants (ASVs). The ASV datasets were normalized by rarefaction to 50,000 reads per sample for diversity analysis. Measures of α-diversity represented by the number of observed ASVs and the Shannon index demonstrated that the diversity of bacterial communities gradually decreased from the bulk soil to the endosphere at 4 WAP, the initial vegetative stage (Fig. 4). This diversity gradient was also observed in other plants such as tomato and rice^32,33^. Although the diversity of bacterial communities in the rhizoplane was temporally decreased during plant development, that in the endosphere was increased; accordingly, the diversity gradient was reversed between the rhizoplane and endosphere at 11WAP, the later reproductive stage (Fig. 4). The diversity of bacterial communities in the endosphere tended to be increased by the application of HV (Supplementary Fig. S6), but it was not statistically significant.

**Figure 5:**
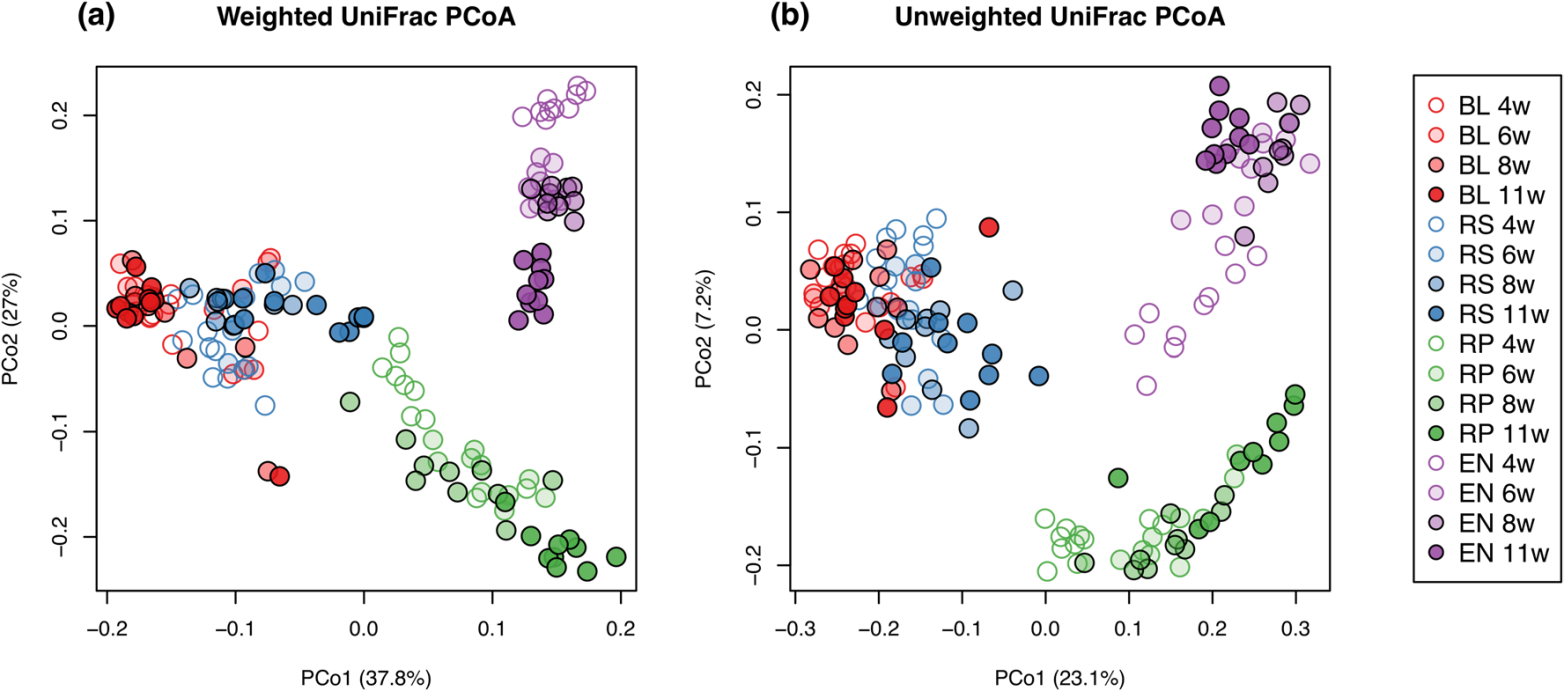
Principal coordinate analysis of weighted and unweighted UniFrac distance among all samples. BL, bulk soil; RS, rhizosphere; RP, rhizoplane; EN, endosphere.

**Figure 6:**
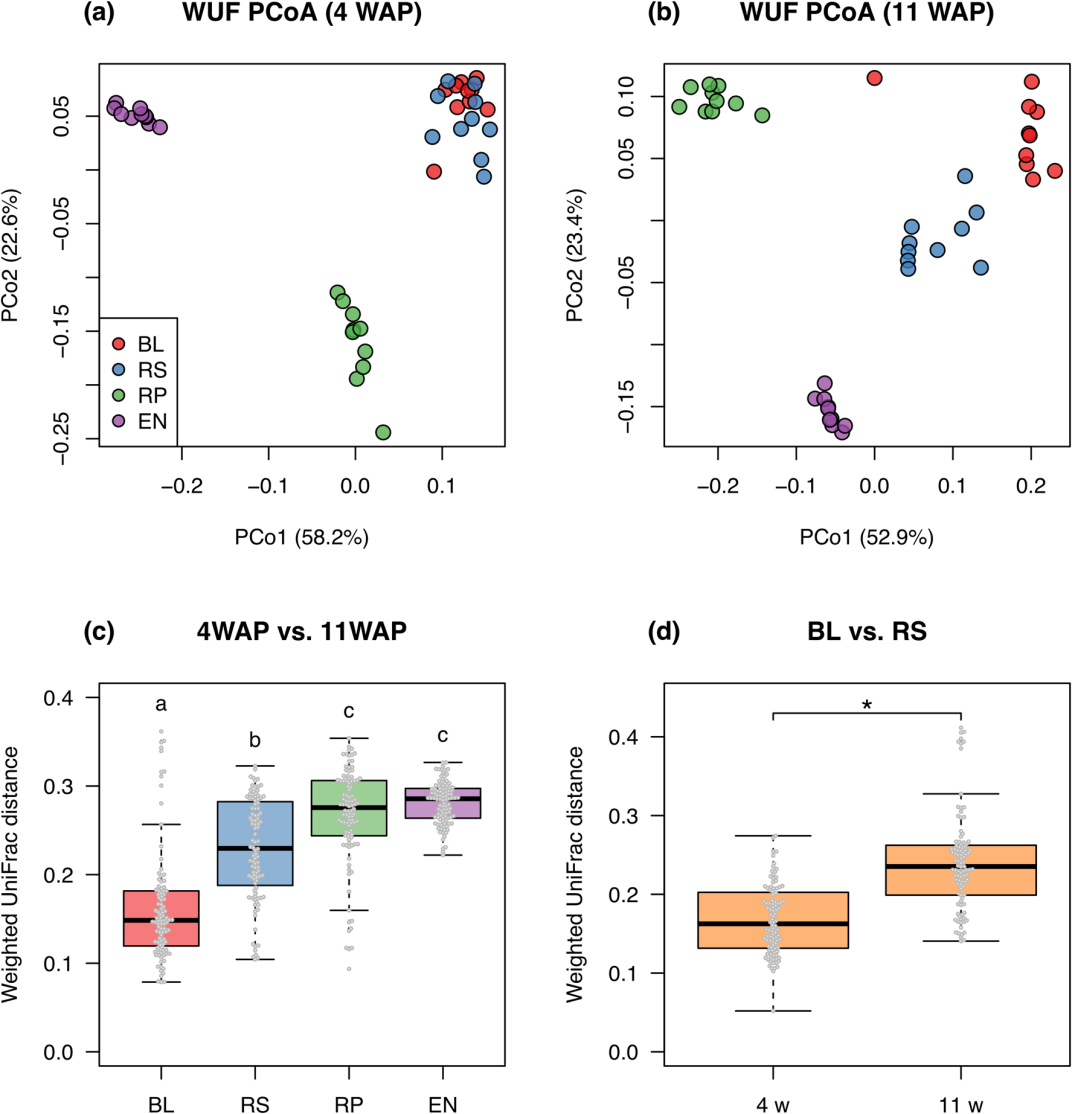
Dissimilarity of bacterial communities between the initial vegetative stage and later reproductive stage. (**a**,**b**) PCoA plots of WUF distance matrices at 4 and 11 WAP, respectively. (**c**) Boxplot of WUF distances between 4 and 11 WAP at each compartment. Different letters above boxes indicate significant differences among different compartments (*p* < 0.05, Wilcoxon rank-sum test with the Bonferroni correction). (**d**) Boxplot of WUF distances between the bulk soil and rhizosphere at 4 and 11 WAP. Asterisk (*) shows significant difference (*p* < 0.05, Wilcoxon rank-sum test). PCoA, principal coordinate analysis; WAP, weeks after planting; WUF, weighted UniFrac; BL, bulk soil; RS, rhizosphere; RP, rhizoplane; EN, endosphere.

To investigate the similarities of bacterial communities among different compartments and different growth stages, we performed the principal coordinate analysis (PCoA) of weighted and unweighted UniFrac (WUF and UUF) distances. WUF PCoA plots of all samples demonstrated that the quantitative community structure was separated by different compartments; specifically, that in the endosphere was most distinct from those in the outside of the roots (Fig. 5a). UUF PCoA plots showed that the qualitative community structure was also separated by compartments, and it could be clearly distinguished into three groups: rhizoplane, endosphere, and others (Fig. 5b). The permutational multivariate analysis of variance (PERMANOVA) based on WUF and UUF distances demonstrated that both community structures were significantly different among compartments (WUF, R^2^ = 0.616, p < 0.001; UUF, R^2^ = 0.290, p < 0.001). In comparison among growth stages, the community structures based on WUF in the rhizosphere, rhizoplane, and endosphere clearly changed during plant development (Supplementary Fig. S7). PERMANOVA confirmed that the community structures were significantly separated by growth stage (Rhizosphere, R^2^ = 0.459, p < 0.001; Rhizoplane, R^2^ = 0.524, p < 0.001; Endosphere, R^2^ = 0.799, p < 0.001), but that in the bulk soil did not dramatically changed (R^2^ = 0.153, p = 0.012). Dissimilarity represented by WUF distances to 4 WAP, the initial vegetative stage, also showed that the community structures in the rhizosphere, rhizoplane, and endosphere significantly shifted in a time-dependent manner (Supplementary Fig. S8). WUF PCoA plot at 4 WAP demonstrated that the community structures were not clearly separated between the bulk soil and rhizosphere (Fig. 6a), but that at 11 WAP, the later reproductive stage, showed clear separation between them (Fig. 6b). Although PERMANOVA described the community structures in the bulk soil and rhizosphere were significantly different at both 4 and 11 WAP (4 WAP, p = 0.002; 11 WAP, p = 0.0012; Additional file 3), those in the rhizosphere changed more drastically during plant development than those in the bulk soil (Fig. 6c) and dissimilarity between the bulk soil and rhizosphere was significantly higher at the later reproductive stage than at the initial vegetative stage (Fig. 6d).

Bacterial communities in HV-treated plants were not significantly different from their controls for any growth stages or compartments (Supplementary Fig. S9; PERMANOVA, p > 0.1; Additional file 3), suggesting that the application of HV had little effect on the root-associated bacterial communities.

**Figure 7:**
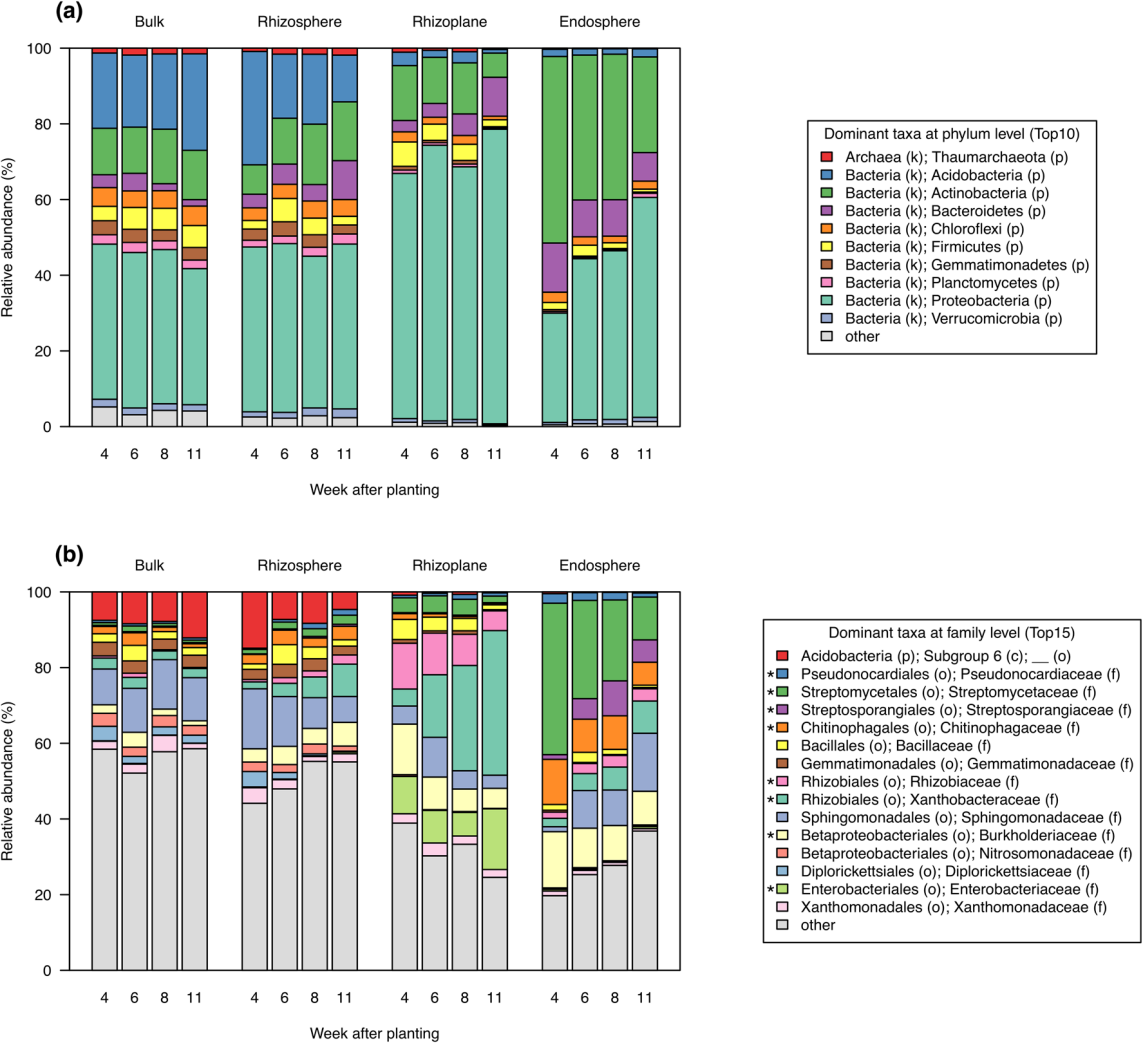
Relative abundance of root-associated bacterial taxa at phylum and family levels. (**a**) Top 10 dominant taxa at phylum level and (**b**) top 15 dominant taxa at family level in root-associated compartment. Asterisks (*) show taxa significantly more abundant in the rhizosphere than in the bulk soil at 11 weeks after planting (FDR < 0.01, ALDEx2). Values are expressed as means of relative frequency at 10 plots.

Phyla distributions and their temporal changes were notably different among the root-associated compartments (Fig. 7a). In the bulk soil, the three most abundant phyla were Proteobacteria, Acidobacteria, and Actinobacteria, and there were no temporal changes in the distribution of these phyla. In the rhizosphere, Proteobacteria was the most abundant phylum, but the relative abundance of Acidobacteria temporally decreased, and that of Bacteroidetes gradually increased. The rhizoplane had a greater proportion of Proteobacteria than the other compartments, and it exhibited temporal decreases in Acidobacteria and Actinobacteria and a temporal increase in Bacteroidetes. In the endosphere, the three most abundant phyla were Actinobacteria, Proteobacteria, and Bacteroidetes, and the proportions of Actinobacteria and Bacteroidetes were gradually decreased and that of Proteobacteria was increased.

Distributions of top 15 dominant taxa at family level were shown in Fig. 7b, and those of top 100 taxa in Supplementary Fig. S10. We performed the differential abundant analysis between the bulk soil and rhizosphere at 11 WAP (Additional file 4). Forty-six taxa were enriched and 13 taxa were depleted in the rhizosphere (Supplementary Fig. S11). Of dominant taxa, eight bacterial taxa including Pseudonocardiaceae (Actinobacteria), Streptomycetaceae (Actinobacteria), Streptosporangiaceae (Actinobacteria), Chitinophagaceae (Bacteroidetes), Rhizobiaceae (Proteobacteria), Xanthobacteraceae (Proteobacteria), Burkholderiaceae (Proteobacteria), and Enterobacteriaceae (Proteobacteria) were significantly more abundant in the rhizosphere than in the bulk soil at the later reproductive stage (Fig. 7b, Supplementary Fig. S12).

### Integrated analysis of soil minerals, microbiome, and transcriptome

**Figure 8:**
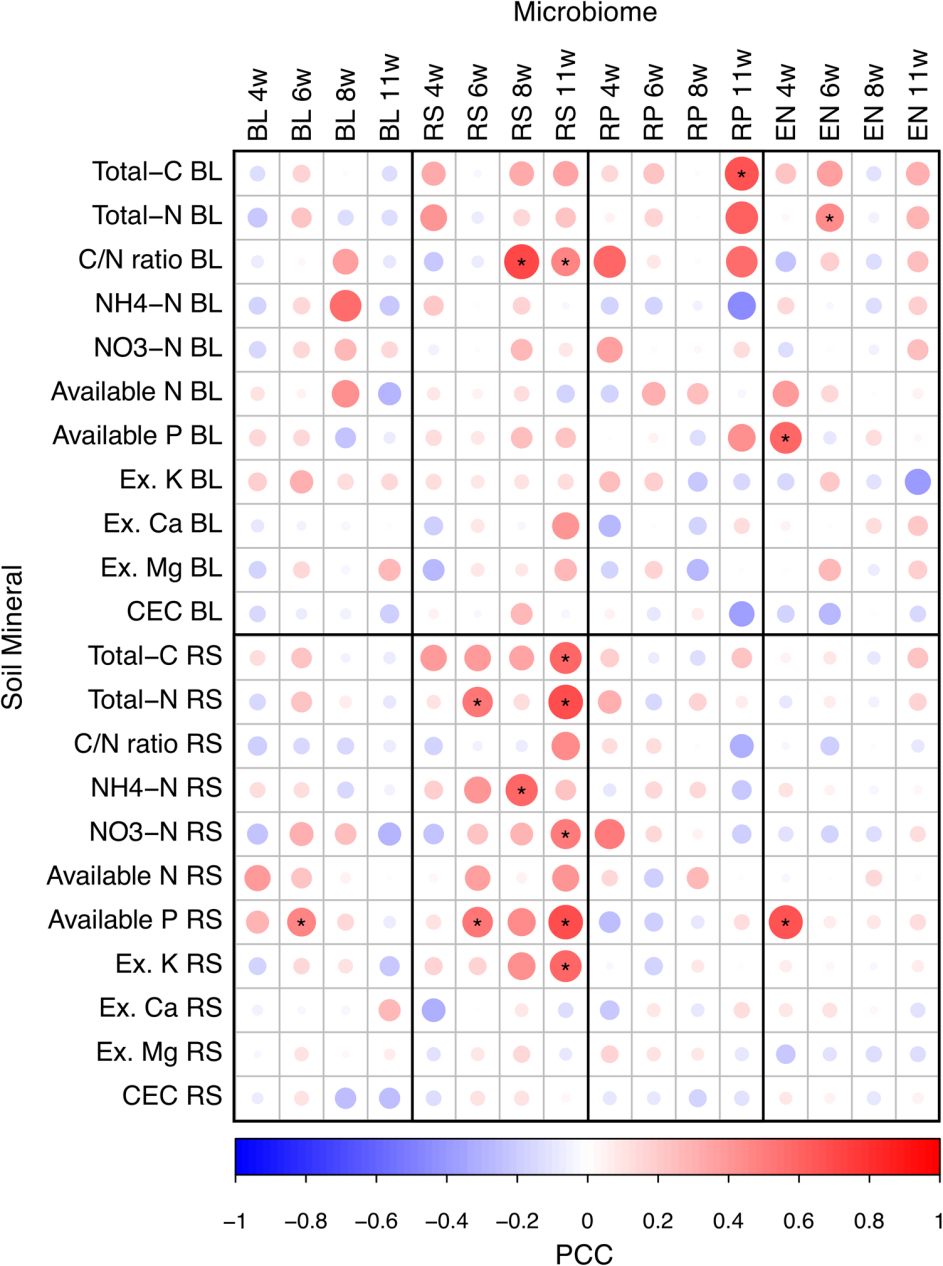
Mantel’s r statistics between root-associated bacterial communities and the soil environment. Mantel’s test was performed using weighted UniFrac (WUF) distance matrix and the Euclidian distance matrix of each mineral contents at each sampling time. Asterisks (*) show significant correlations (*p* < 0.01). PCC, Pearson correlation coefficient; BL, bulk soil; RS, rhizosphere; RP, rhizoplane; EN, endosphere; Ex, exchangeable; CEC, cation exchange capacity.

To investigate the relationships between bacterial communities and environmental factors in the rhizosphere, we used Mantel’s test to compare the WUF distance of bacterial communities with the Euclidian distance of soil mineral contents (Fig. 8). Notably, at 11 WAP in the rhizosphere, several mineral factors such as total C, total N, NO_3_-N, available P, and exchangeable K had significant correlations with the bacterial communities. By contrast, bacterial communities in other compartments showed only few or no correlations with environmental factors in the bulk soil and rhizosphere. This indicated that the bacterial community and mineral environments interact with each other, especially in the rhizosphere.

**Figure 9:**
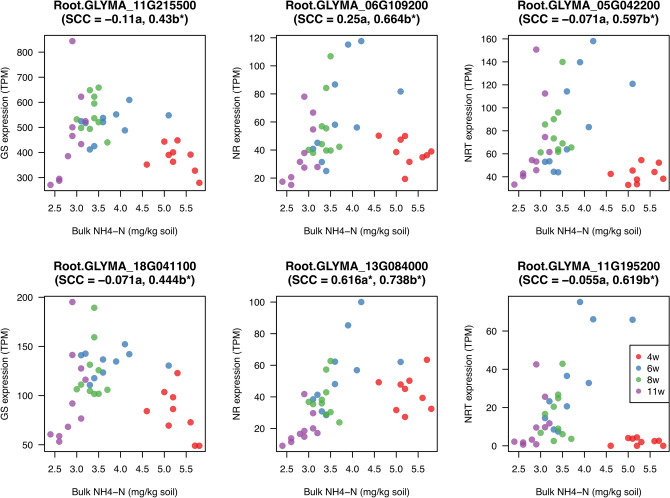
Correlation between the expression of nitrogen-responsive genes and the content of NH_4_–N in the bulk soil. Scatter plots of gene expression levels and NH_4_–N contents, and Spearman correlation coefficients (SCC). SCC values with “a” indicate correlation during 4–11 WAP and those with “b” during 6–11 WAP. Asterisks (*) show significant correlations (*p* < 0.05).

Mantel’s test was also performed for the correlation matrix of plant transcriptome and the Euclidian distance of soil mineral contents (Supplementary Fig. S13). However, only a few mineral factors in the rhizosphere were significantly correlated with gene expression profiles. To evaluate the plant physiological response to the field environment, we examined the correlation between gene expression in the soybean and mineral contents in the soil. Expression of nitrogen-responsive genes such as glutamine synthetase (GS; Glyma.11g215500, Glyma.18g041100), nitrate reductase (NR; Glyma.06g109200, Glyma.13g084000), and nitrate transporter (NRT; Glyma.05g042200, Glyma.11g195200) were positively correlated with the content of inorganic nitrogen such as NH_4_-N and NO_3_-N in the bulk soil (Fig. 9, Supplementary Fig. S14) during 6 to 11 WAP, but not in the rhizosphere soil (Supplementary Figs. S15, S16). Gene expression levels at 4 WAP were relatively low and did not show a significant correlation, probably because of its small plant body. This result suggests that the mineral environment in the bulk soil affected the physiological status in the soybean plants.

**Figure 10:**
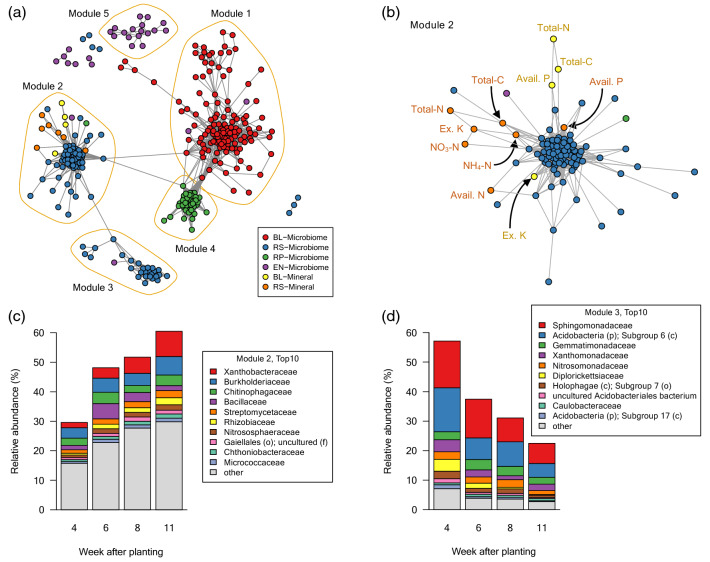
Co-abundance network among soil minerals and bacterial taxa at the family levels in root-associated compartments. (**a**) Co-abundance network constructed based on the correlation. Four networks in four growth stages were merged, and the consistent network at all growth stages were extracted. Modules were found based on the fast greedy modularity optimization algorithm. Nodes represent bacterial taxa at the family level and mineral components, and edges are drawn based on the topological overlap. (**b**) Sub-network of the Module 2. (**c**) Relative abundance of taxa belonging to the Module 2 in the rhizosphere. (**d**) Relative abundance of taxa belonging to the Module 3 in the rhizosphere. BL, bulk soil; RS, rhizosphere; RP, rhizoplane; EN, endosphere; Avail, available; Ex, exchangeable.

We constructed a co-abundant network among minerals and microbial taxa at the family level in each root-associated compartment. To extract robust relationships during plant development, the network figures in four growth stages were merged, and the intersection of the networks at all growth stages was shown in Fig. 10. The microbial taxa in each compartment were highly clustered, and five modules in the network were found based on the fast greedy modularity optimization algorithm^34^. Each module almost corresponded to each compartment, and microbial taxa in the rhizosphere formed two modules, Module 2 and Module 3 (Fig. 10a; Additional file 5). In the Module 2, we found that the minerals such as total C, total N, NH_4_-N, NO_3_-N, available N, available P, and exchangeable K in the rhizosphere were correlated with the microbial taxa in the rhizosphere (Fig. 10b), but not in the bulk soil in the Module 1. Relative abundance of the taxa composing the Module 2 gradually increased in the rhizosphere during plant development (Fig. 10c), contrasting to the gradual decrease of the taxa belonging to the Module 3, that was not connected to any minerals (Fig. 10d). These results are consistent with the significant relationship between the bacterial communities and the mineral contents in the rhizosphere as shown in Fig. 8, and it also suggests that the bacteria and the minerals in the rhizosphere are closely interrelated with each other during plant development.

## Discussion

The root-associated environments are an important soil region that directly affects plant growth and crop production and reflects the host plant's status. Many studies have performed the characterization of those environments on the individual aspects of biological and chemical properties. However, for a comprehensive understanding of the root-associated environments, the integration of multiple layers of data is needed. In the present study, we performed a multi-omics analysis of the root-associated environments of soybean grown in the field and analyzed the interaction between biological and chemical components.

Our field experiment was designed to explore the effect of HV application on the rhizosphere environment including microbiota and mineral nutrients as well as the effect on the soybean physiological states. HV is a leguminous cover crop that improves soil properties and increases crop production^28,29^, and it also has an allelopathic activity^35–37^. Allelochemicals derived from allelopathic plants have effects not only on surrounding plants but also on soil microbial populations and communities^38,39^. We therefore expected HV to affect the rhizosphere environment. An increase in NO_3_-N content in the bulk soil and slight promotion of soybean growth with HV application (Fig. 1, Supplementary Fig. S4) indicated that HV indeed improved soil fertility in our field experiment. However, we observed almost no effects on the root-associated microbiome and gene expression of soybean (Supplementary Fig. S9, Additional file 1), probably because the field soil was rich in organic matter and mineral nutrients by application of fertilizer until the previous year. Allelopathic activity in the soil is affected by various soil factors including organic matter^40^, and symbiotic nitrogen fixation in legumes is suppressed by nitrogen fertilizer^41,42^.

Transcriptome analysis of soybean plants grown in the field revealed that the expression of genes associated with nitrogen assimilation in the root was positively correlated with inorganic nitrogen contents in the bulk soil (Fig. 9, Supplementary Fig. S14). In *Arabidopsis*, the glutamine synthetase gene *GLN1;2* is upregulated by ammonium and nitrate supply^43,44^. Expression of nitrate reductase genes *NR1* and *NR2* and the nitrate transporter genes *NRT1.1* and *NRT2.1* is also induced by the presence of nitrate^45–47^. Although short-term induction of these genes was observed in *Arabidopsis* in laboratory experiments^45–47^, our data demonstrated that long-term responses at the basal expression level were present in soybean plants grown in the field.

Gene expression patterns at different growth stages of soybeans in the field were partly consistent with those in the laboratory^31^. Upregulation of genes responsive to biotic stress (chitin and fungus) and downregulation of genes related to photosynthesis in the leaves at a later stage were commonly observed in the laboratory^31^ and the field (Fig. 3), indicating that those gene expression patterns are typical responses to leaf senescence in the soybean plants^31^. Upregulation of signaling pathways mediated by jasmonic acid, salicylic acid, and abscisic acid at a later stage (Fig. 3) are consistent with these plant hormones promoting leaf senescence^48^. In contrast to that of the leaf, the senescence process of the root is poorly understood^49^. Our data suggest that the downregulation of genes related to cell wall biosynthesis in the roots at a later stage (Fig. 3) may represent root senescence or suppression of root elongation.

Microbiome analysis characterized the spatial and temporal dynamics of root-associated microbiota of soybean plants under field conditions. Bacterial communities in the rhizosphere changed more robustly during soybean development than those in the bulk soil (Fig. 6c, Supplementary Fig. S7), which is consistent with a previous report^50^. Additionally, our analysis demonstrated that bacterial communities in the rhizoplane and endosphere also drastically changed (Fig. 6c, Supplementary Figs. S7, S8). Previous studies reported that root microbiota in rice was highly dynamic at the vegetative stage and then stabilized at the reproductive stage^51^. We also found such dynamic changes of microbiota in soybeans at the vegetative stage (Supplementary Figs. S7, S8), which is consistent with the high secretion of daidzein from the soybean root at the vegetative stage^52^. Daidzein is an isoflavone involved in shaping rhizosphere microbiota and enriches specific bacterial taxa such as Comamonadaceae^18^. Such enrichment of Comamonadaceae (assigned as Burkholderiaceae on the database we used) in the rhizosphere was also observed in our analysis (Fig. 7, Supplementary Fig S12), suggesting that it is a consistent modification in the soybean rhizosphere even at different locations. By contrast, our analysis revealed that bacterial communities in the rhizosphere, rhizoplane, and endosphere were also significantly shifted during the reproductive stage (Supplementary Figs. S7, S8). Thus, different metabolites may be involved in microbiome acquisition at different growth stages of soybean plants in the field, and additional investigation including metabolome in the rhizosphere will further develop our understanding.

Soil mineral analysis using a small-scale protocol^30^ showed that most of the minerals, especially NH_4_-N and exchangeable K, accumulated to high concentrations in the soybean rhizosphere (Fig. 2), which was defined as the soil adhering to the root surface even after shaking (see Materials and Methods). This is consistent with other plant rhizospheres such as sorghum (*Sorghum bicolor*) measured through the same method^30^. By contrast, previous rhizobox experiments with separation of soil surrounding the roots into small compartments showed that those nutrients exhibited a decreasing gradient from the surrounding soil to the root surface^22,53^. These apparent inconsistent results may indicate that proximity to the root surface has a great effect on mineral content around the roots.

Mantel’s test and network analysis among bacterial communities and mineral contents revealed that the bacterial communities in the rhizosphere were significantly interrelated with mineral content in the rhizosphere (Figs. 8, 10). In particular, the unique bacterial communities to the rhizosphere at the later reproductive stage (Fig. 6b) significantly interacted with several mineral components (Fig. 8). Furthermore, the bacterial taxa enriching in the rhizosphere (Fig. 10c) were correlated with minerals at all growth stages (Fig. 10b). This suggests that the specific bacterial communities in the rhizosphere affect the availability of mineral nutrients and consequently may have significant effects on plant growth. In the Module 2 in the co-abundant network (Fig. 10b), some of the families such as Rhizobiaceae, Chitinophagaceae, and Streptomycetaceae include strains previously isolated as plant growth-promoting rhizobacteria (PGPR)^54–59^, and they were also dominant bacterial families in the rhizosphere at the later reproductive stage (Fig. 7b). PGPR generally affect plant growth directly by biological nitrogen fixation, phosphate solubilization, and phytohormone production and indirectly by biocontrol of pathogen and induction of plant resistance^60–62^. Some isolates belonging to Rhizobiaceae or Chitinophagaceae are reported to solubilize phosphate, produce auxin, and increase plant biomass^54–57^. Some members of *Streptomyces* produce antibiotics and prevent pathogen infection in host plants^58,59^. *Streptomyces* also is an ammonifying bacteria that decomposes organic nitrogen compounds into ammonia in the soil^63,64^. Other PGPR strains belonging to Bacillaceae, that is included in the Module 2 in our network (Fig. 10b,c), are also reported to solubilize potassium and increase its uptake into plants^65,66^. These are consistent with our network in that the Module 2 include minerals such as available P, NH_4_-N, and exchangeable K in the rhizosphere (Fig. 10b). Together with these previous reports, our findings suggest that specific bacterial activities in the rhizosphere may cause great accumulation of minerals such as NH_4_-N and exchangeable K in the rhizosphere (Fig. 2).

In summary, we performed a field multi-omics analysis and characterized the spatiotemporal dynamics of the soybean root-associated environment in the actual field condition. Mineral contents and bacterial communities in the rhizosphere were distinct from those in the bulk soil, and they significantly interacted with each other. Our findings suggest that the rhizosphere bacterial community has a close association with mineral availability in the rhizosphere. Further understanding of the root-associated environment and optimal modification of them will achieve the robust crop production under environmental changes.

## Materials and methods

### Plant materials, cultivation schedule, and sampling

Soybean cultivation was conducted at the experimental field of the Tokyo University of Agriculture and Technology, Japan (35°40′59.2" N, 139°29′05.3" E), in 2017–2018. Ten plots (4 × 10 m) within the farm were established for the current experiment. In each of the odd-numbered plots (1, 3, 5, 7, 9 plot), a winter cover crop, HV (*Visia villosa* Roth subsp. Villosa, Takii) was hand sown in early November 2017. The even-numbered plots (2, 4, 6, 8, 10 plot) were kept as fallow. The field was maintained organically without the addition of fertilizer. HV and native weeds were allowed to grow until May 2018. The soil was then plowed to a depth of about 30 cm to incorporate cover crop residues (plant organic materials) using a rotary machine. Control plots were also tiled but with no additional plant materials. The summer crop, soybean seeds (*Glycine max*, cv. Enrei, Takii Shubyo, Japan) were sown using a seeding machine in June 2018. For analysis of bacterial communities, six soybean plants from each plot were collected on July 4, 2018 (early vegetative stage), July 18, 2018 (beginning bloom stage), August 1, 2018 (full bloom stage), and August 21, 2018 (beginning seed stage). Bulk soil, defined as soil that is at least 20 cm from the plants and does not adhere to plant roots^50^, was also collected at this time. The bulk soil in each plot was obtained by removing the top 5 cm of the surface soil and then collecting the underneath soil using a shovel. The soil was collected from five locations in each plot and then combined. For gene expression analysis of soybean plants, five lateral roots and one top leaf from five plants in each plot were collected and immediately frozen in the field with dry ice. For evaluation of plant growth, a portable SPAD-502 plus chlorophyll meter (Konica Minolta Company, Japan) was used to measure relative chlorophyll content in soybean (SPAD value)^67^. The chlorophyll content of the top three fully expanded leaves of each plant was measured and the average SPAD value was recorded. This procedure was repeated for three plant in each plot in the farm condition.

### Sample preparation of root-associated compartments

Rhizosphere and rhizoplane soil were collected as described previously^50,68^. Plant roots were shaken vigorously to remove any excess soil. The soil still adherent to the root was gently brushed off onto clean paper using a brush and sieved with a tea strainer (1 mm × 1 mm mesh) to remove excess organic material and gravels. The sieved soils were considered to be “Rhizosphere soil.” Roots used for collecting rhizosphere soil were subsequently used for collection of “Rhizoplane soil.” The roots were put in a 500 mL beaker, and 300 mL potassium phosphate saline buffer (PBS) containing surfactant (0.1% Silwet L-77) was added to fully submerge the roots. All beakers were placed on a rotary shaker and shaken for 10 min to remove the excess soil particles and organic material from the root. The roots were put in clean beakers with buffer and placed in a sonic bath at 25 °C for 10 min. The solution was then centrifuged at 5,000 × *g* for 15 min, and the precipitation was collected as Rhizoplane soil. For collection of the roots used for extraction of endophytes, four healthy, lateral roots of each plant were selected after the rhizoplane soil was collected. The lateral roots were cleaned of rhizobium. All samples were immediately stored at − 80 °C until further use.

### Soil mineral analysis

Mineral contents were measured using a set of small-scale protocols for analyzing the nutrient minerals of small soil samples as described previously^30^. Briefly, total C and total N contents were measured using an NC analyzer (Sumigraph NC-22F; Sumika Chemical Analysis Service, Ltd., Osaka, Japan). The available N content was estimated using the phosphate buffer extraction of soil organic N. Inorganic N (NH_4_-N, NO_3_-N) was extracted using a potassium sulfate solution and measured by colorimetric analysis. The available P content was estimated using the Truog method: extraction using a weak acidic solution and measurement by colorimetric analysis. Exchangeable bases (K, Ca, Mg) were extracted using an ammonium acetate solution and measured by flame photometry for K and by atomic absorption spectrometry (AA-6200; Shimadzu, Kyoto, Japan) for Ca and Mg. CEC was measured using residual soil after extraction of exchangeable bases, and ammonium ion was used as an exchanger cation.

### Transcriptome

Soybean tissues were homogenized using a pestle and mortar pre-chilled in liquid nitrogen. Total RNA was isolated using the RNeasy Plant Mini Kit (QIAGEN, Hilden, Germany) with in-column DNase I digestion according to the manufacturer’s protocol. The RNA samples were sequenced by single-end 50 bp mode of HiSeq2500 platform (Illumina, CA).

RNA-seq reads were aligned to the soybean genome assembly v2.1^69^ using STAR v2.7.0f.^70^ based on Ensembl Plants release 43^71^ gene annotations. Gene expression level was estimated as transcripts per million (TPM)^72^ using RSEM v1.3.0^73^ with default parameters.

After filtering low-expression genes based on the distribution of expression levels (Supplementary Fig. S17), the whole transcriptome data set (41,682 genes; TPM > 0.125) was used for PCA analysis. To identify differentially expressed genes, we used the count data of 36,516 genes (TPM > 0.125) in the leaf and 39,028 genes (TPM > 0.25) in the root, and the R package DESeq2^74^ with a false discovery rate (FDR) of 0.05 and twofold change. Gene expression profiles were classified into six patterns: significant increase or decrease only between 4 and 6 WAP (early stage), 6 and 8 WAP (middle or mid stage), or 8 and 11 WAP (later stage). GO enrichment analysis was carried out using the Soybase GO term enrichment tool (https://www.soybase.org).

### Microbiome

DNA was extracted from 250 mg of the soil samples with a DNeasy PowerSoil Kit (QIAGEN, Hilden, Germany). To crush bacterial cells, a milling machine (CellDistroyer, Pro Sense Inc, model PS100) was used. For extraction of endophytic bacterial DNA from soybean roots, the same kit was also used. The extracted DNA was quantified using the Quantus Fluorometer and QuantiFluor Dyes (Promega Corporation, Madison, CA) and stored at − 80 °C until use.

PCR amplification of the V4 region of 16S rRNA genes was performed as described previously^18^. Each 25 μL of reaction mixture contained 1 ng template DNA, 0.3 μL of KOD FX neo (Toyobo, Osaka, Japan), 12.5 μL of buffer (provided with the polymerase), 5 μL of dNTPs (2 mM), and 0.75 μL of 515F (5′-ACACTCTTTCCCTACACGACGCTCTTCCGATCT-GTGCCAGCMGCCGCGGTAA-3′) and 806R (5′-GTGACTGGAGTTCAGACGTGTGCTCTTCCGATCT-GGACTACHVGGGTWTCTAAT-3′) primers. PCR conditions were as follows: initial denaturation at 94 °C for 2 min and 22 cycles at 98 °C for 10 s, 50 °C for 30 s, and 68 °C for 30 s. The PCR products were purified using AMPure XP magnetic beads (Beckman-Coulter, Indianapolis, IN, USA). For the amplification of bacterial endophytic DNA, 2.5 pmol/μL peptide nucleic acid (Fasmac Co., Ltd, Kanagawa, Japan) were added to the reaction mixture to avoid the amplification of root mitochondrial DNA.

To attach MiSeq adaptors, a second round of PCR was performed in a 25 μL reaction mixture containing 2 μL template DNA (purified from the first PCR product), 0.3 μL of KOD FX neo (Toyobo, Osaka, Japan), 12.5 μL of buffer (provided with the polymerase), and 0.75 μL of primers provided with Fasmac Co. Ltd. The second PCR products were purified using AMPure XP magnetic beads and confirmed by electrophoresis on 1.5% agarose gels. The DNA concentration was measured using the Quantus Fluorometer and QuantiFluor Dyes (Promega Corporation, Madison, CA, USA) according to the manufacturer’s protocol. Amplicon sequencing using the Illumina MiSeq platform (2 × 250 bp) was outsourced to Fasmac Co., Ltd.

The obtained sequences were processed and analyzed using the QIIME2 pipeline (version 2019.10)^75^. Raw fastq files were imported into QIIME2, and paired-end sequences were trimmed at the first 20 bases, truncated at 200 bases from the start, quality filtered, denoised, and merged using DADA2^76^ with the q2-dada2 plugin in QIIME2. Multiple alignments of the representative sequences were performed using the MAFFT program, and the phylogenetic tree was generated with the FastTree program in the q2-phylogeny plugin^77,78^. Taxonomy was assigned to the sequences using the q2-feature-classifier plugin and a Naive Bayes classifier, which was pre-trained on operational taxonomic units (99% identity) from 515F/806R region of sequences on the SILVA rRNA gene database release 132^79,80^. After filtering mitochondrial and chloroplastic sequences, 52,780–321,718 reads per sample were obtained. Alpha and beta diversity analysis was performed using the core-metrics-phylogenetic pipeline in the q2-diversity plugin within QIIME2, which rarefies ASV tables to 50,000 reads and calculates Shannon’s diversity index, observed ASVs, Faith’s phylogenetic diversity, and Evenness for alpha diversity. It furthermore computes Jaccard, Bray–Curtis, and weighted and unweighted UniFrac distances for beta diversity and generates PCoA plots for each beta diversity metrics. Association between categorical metadata groups and alpha diversity metrics were tested by Kruskal–Wallis and corrected by the Benjamini–Hochberg method, and that for beta diversity were analyzed by PERMANOVA and corrected by the Benjamini–Hochberg method using the R software packages stats and vegan^81^.

To identify differential abundant bacterial taxa at the family level between in the bulk soil and rhizosphere, we used the R software package ALDEx2^82,83^ with an FDR cutoff of 0.01.

### Mantel’s test

Mantel’s test was performed to investigate the relationship between bacterial community structure and soil chemical property. We used the weighted UniFrac distance of the bacterial community within a compartment and the Euclidian distance of each mineral content. The correlations between those distance matrices were analyzed using the R software package vegan^81^ with 10,000 permutations.

The correlation between gene expression profile and soil chemical property also was analyzed by Mantel’s test. We calculated the correlation distance matrix of gene expression profiles as the subtraction of one from a correlation matrix of whole transcriptome data sets without low expression genes.

### Network analysis

A singed correlation network was constructed among the abundance of bacterial taxa at the family level and concentrations of minerals using the weighted gene co-expression network analysis (WGCNA) with the R package WGCNA^84^. Count data of taxonomy profile were log-transformed using the R packages phyloseq^85^ and microbiome^86^, and low abundant taxa (Max < 0.1%) from each compartment at each sampling time were filtered out. The soft thresholding power for each dataset at four growth stages was chosen based on the scale-free topology fit index of 0.85. The adjacency matrix was calculated using the soft thresholding power, and it was transformed into the topological overlap matrix. Network connections whose topological overlap was above the thresholds of 0.05 were extracted. Networks at four growth stages were merged, and intersection of them was visualized using the R package igraph^87^.

### Data visualization

Statistical analysis was conducted with the R software^88^. Figures are drawn using the R packages gplots^89^, corrplots^90^, and beeswarm^91^.

## Supplementary Information

Supplementary Information 1.

Supplementary Information 2.

Supplementary Information 3.

Supplementary Information 4.

Supplementary Information 5.

Supplementary Information 6.

## Data Availability

The accession numbers of sequence data has been registered in the DNA Data Bank of Japan (DDBJ) Sequence Read Archive are DRA011419 and DRA011795.
