# Supplementary material for: Field multi-omics analysis reveals a close association between bacterial communities and mineral properties in the soybean rhizosphere
Source: Sci Rep. 2021 Apr 23;11:8878. doi: 10.1038/s41598-021-87384-8 (PMC8065045; doi:10.1038/s41598-021-87384-8)
Supplement: Supplementary file 6 — Supplementary Information 6. [file 41598_2021_87384_MOESM6_ESM.pdf]

Title

Field multi-omics analysis reveals a close association between bacterial communities and mineral properties in the soybean rhizosphere

Authors

Shinichi Yamazaki[1†], Hossein Mardani-korrani[2†], Rumi Kaida[2†], Kumiko Ochiai[3], Masaru Kobayashi[3], Atsushi J. Nagano[4], Yoshiharu Fujii[2], Akifumi Sugiyama[5], Yuichi Aoki[1*]

Affiliations

1. Tohoku Medical Megabank Organization, Tohoku University, Sendai, Japan
2. Department of International Environmental and Agricultural Science, Tokyo University of Agriculture and Technology, Fuchu, Japan
3. Division of Applied Life Sciences, Graduate School of Agriculture, Kyoto University, Kyoto, Japan
4. Faculty of Agriculture, Ryukoku University, Otsu, Japan
5. Research Institute for Sustainable Humanosphere, Kyoto University, Gokasho, Uji, Japan

† These authors contributed equally to this work.

* Corresponding author

E-mail: aokibluetree@gmail.com

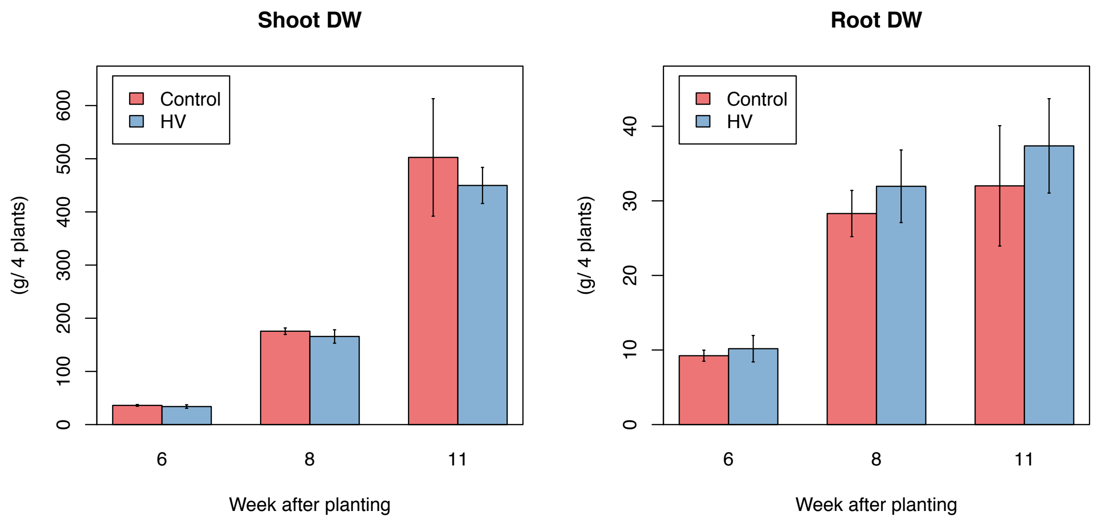

Supplementary Figure S1. Growth of soybean.

Values are expressed as means ± SD (n = 5). Asterisks (*) show significant differences between the control plots and HV plots ($p < 0.05$, t-test). DW, dry weight; HV, hairy vetch.

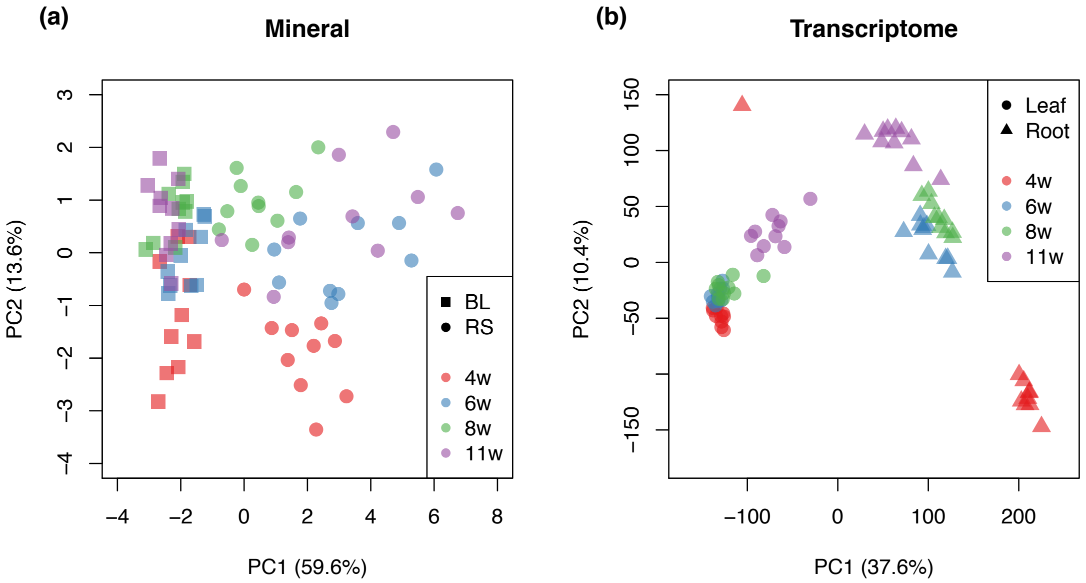

Supplementary Figure S2. Principal component analysis of mineral contents and transcriptome data set.

BL, bulk soil; RS, rhizosphere.

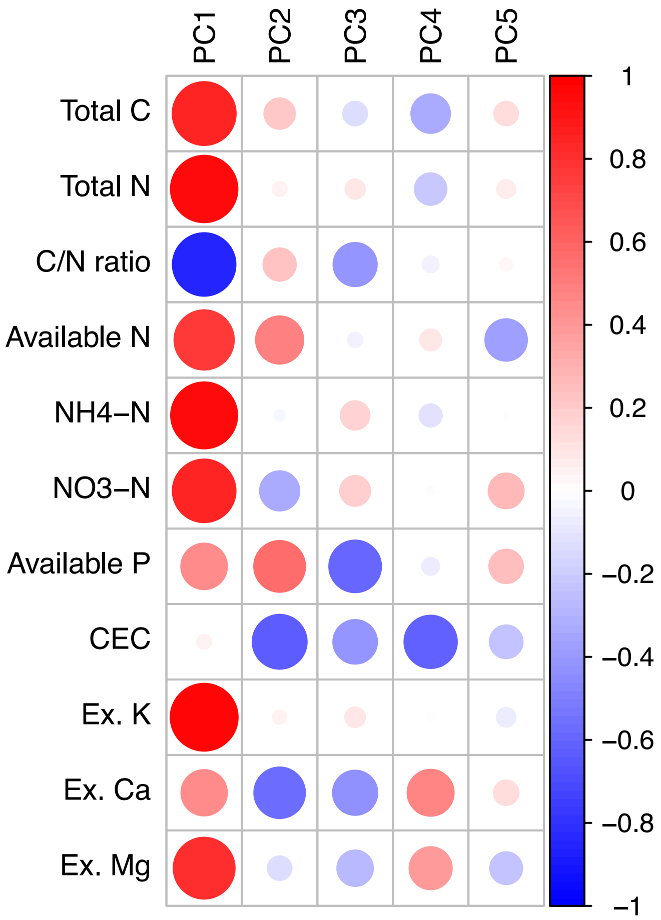

Supplementary Figure S3. Loadings in PCA of mineral contents.

CEC, cation exchange capacity; Ex, exchangeable.

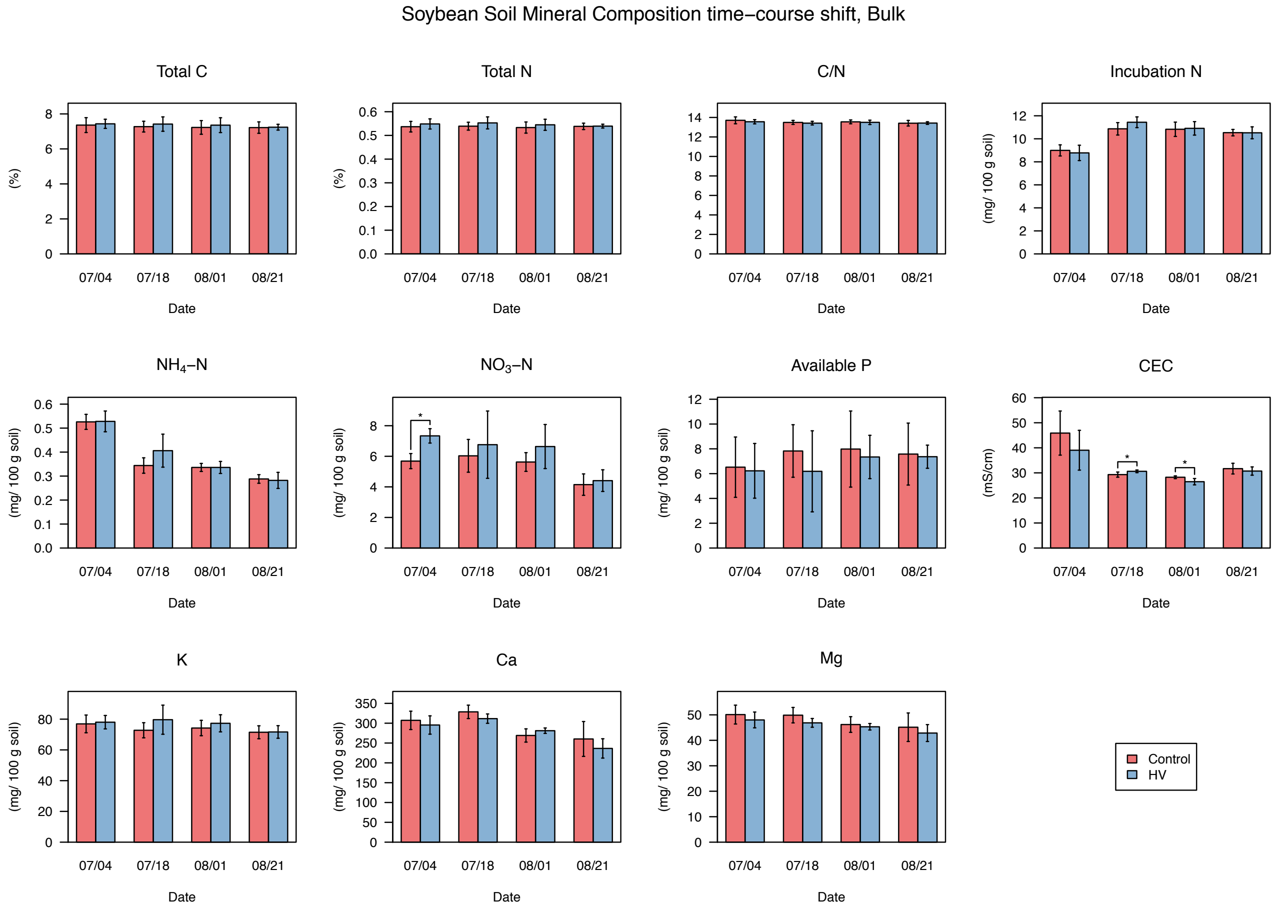

Supplementary Figure S4. Effect of hairy vetch application on the mineral contents in the bulk soil.

Values are expressed as means ± SD (n = 5). An asterisk (*) shows a significant difference between the control plots and HV plots ($p < 0.05$, t-test). HV, hairy vetch; CEC, cation exchange capacity; Ex, exchangeable.

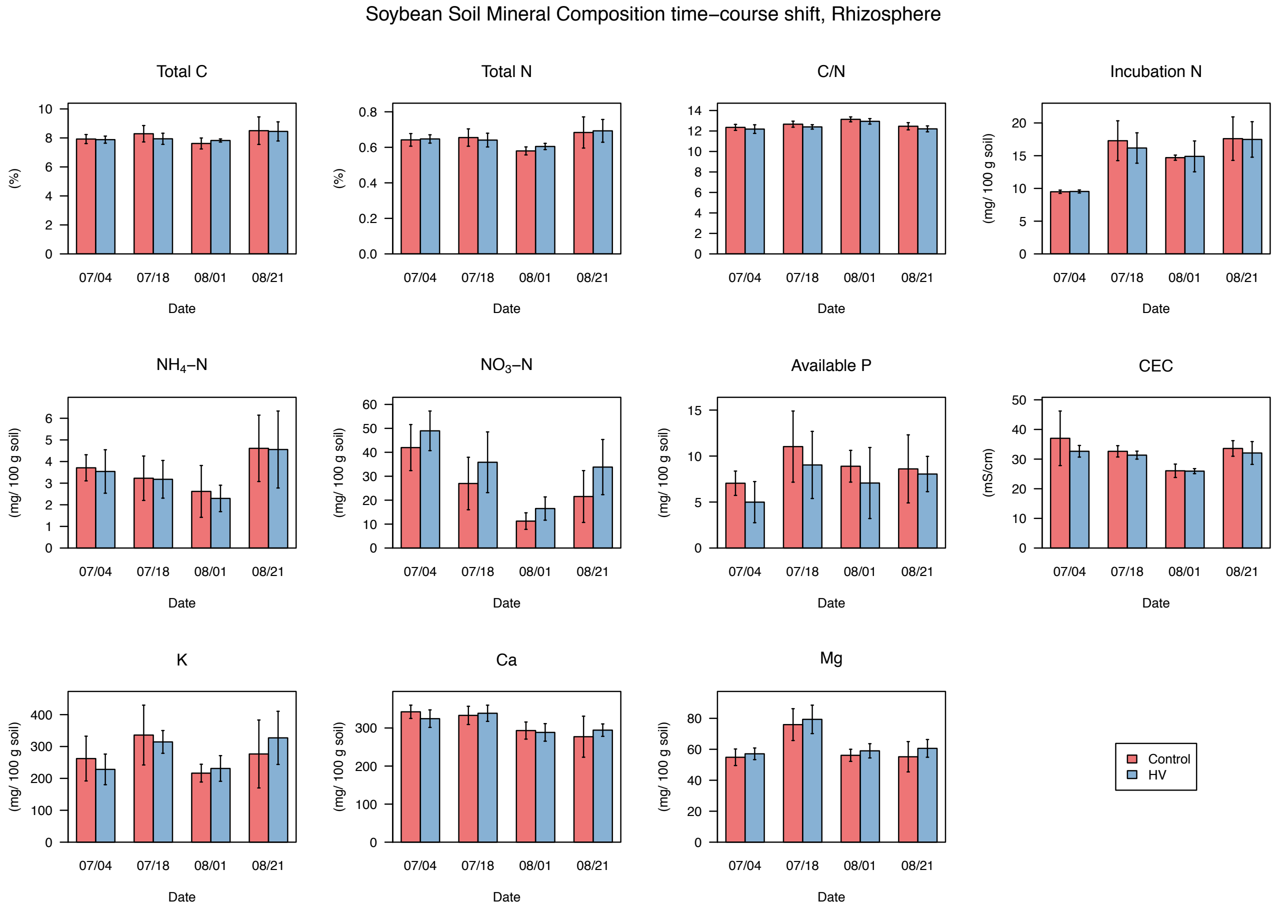

Supplementary Figure S5. Effect of hairy vetch application on the mineral contents in the rhizosphere soils of soybean.

Values are expressed as means ± SD (n = 5). There is no significant difference between the control plots and HV plots ($p > 0.05$, t-test). HV, hairy vetch; CEC, cation exchange capacity; Ex, exchangeable.

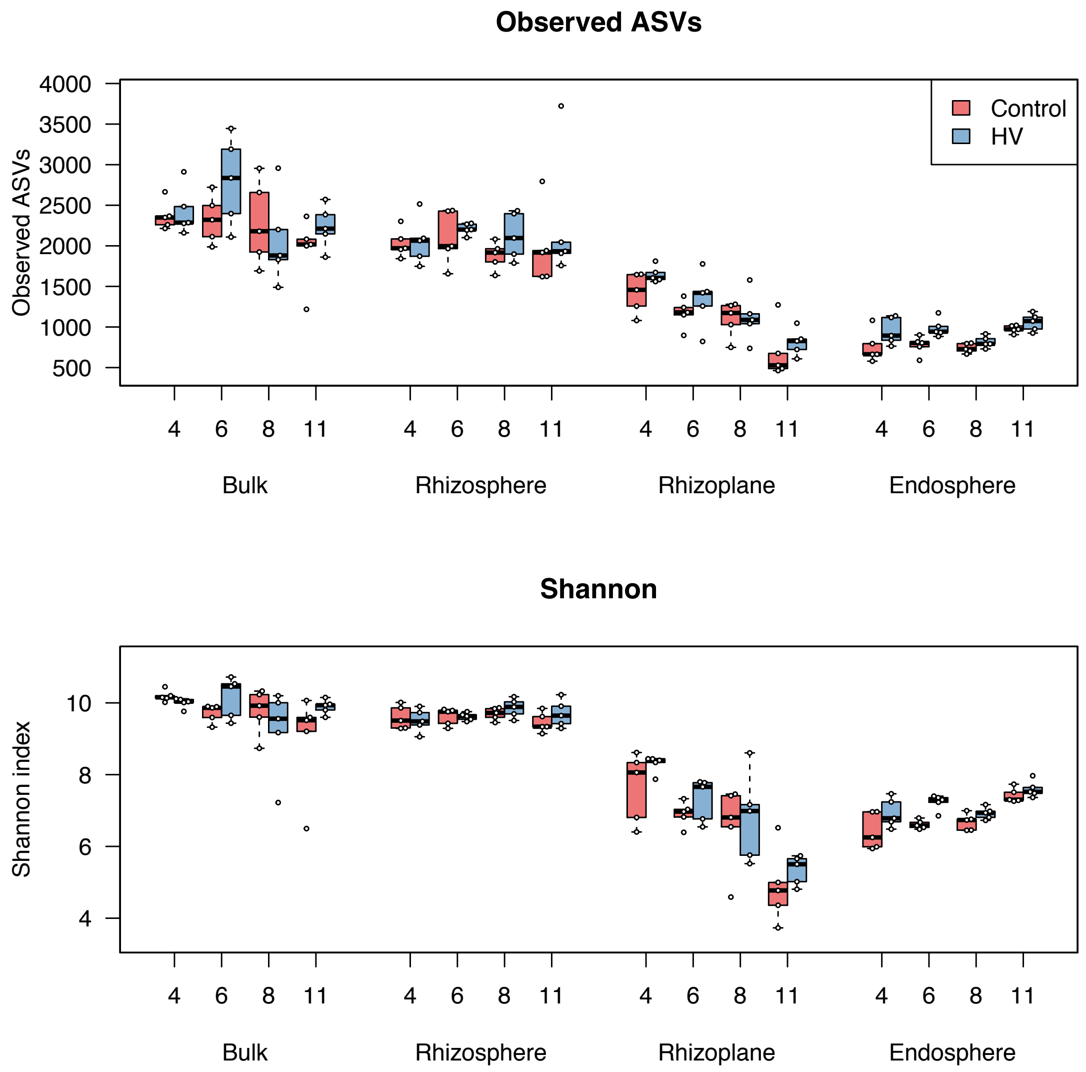

Supplementary Figure S6. Effect of hairy vetch application on the alpha diversity of root-associated bacterial communities.

There is no significant difference between the control plots and HV plots ($p > 0.05$, Kruskal-Wallis test). HV, hairy vetch.

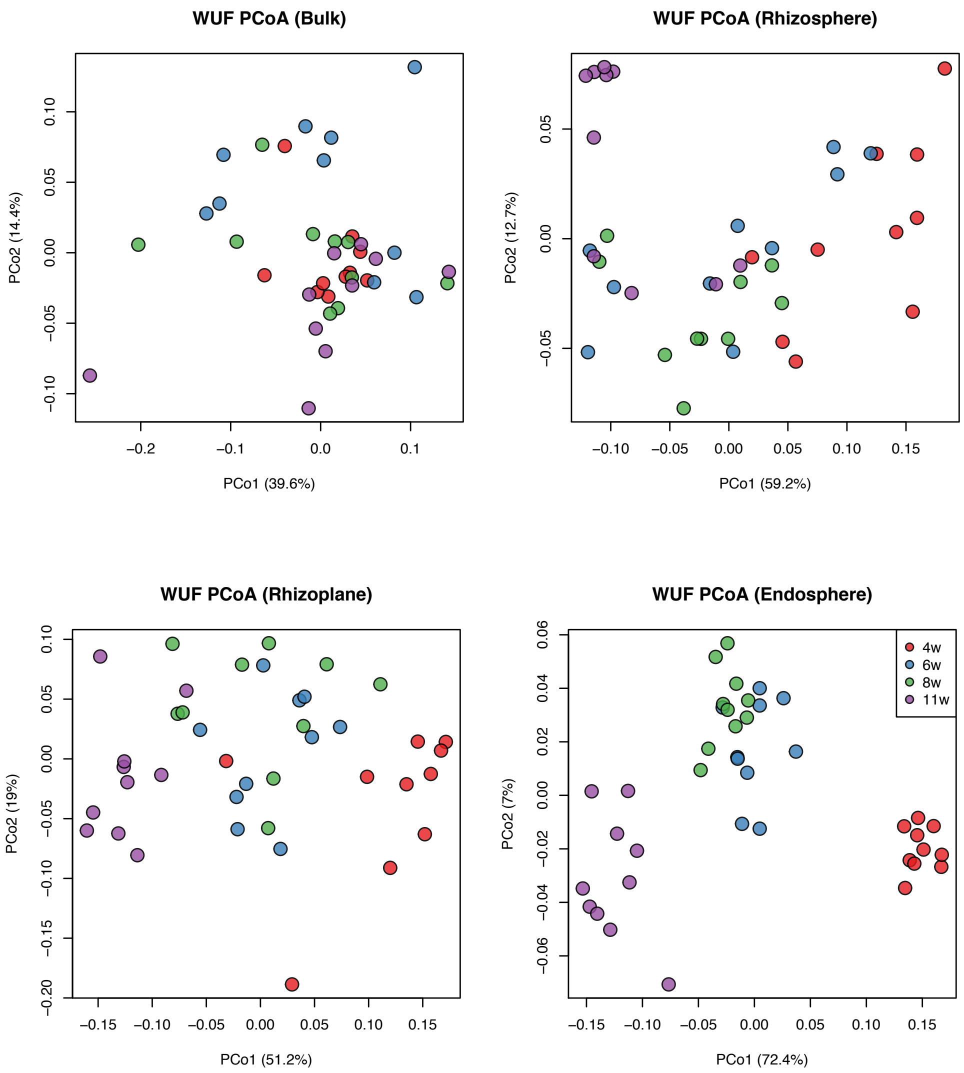

Supplementary Figure S7. Time-course shifts of bacterial communities in root-associated compartments during plant development.

The principal coordinate analysis (PCoA) of weighted UniFrac distances were performed. Results of the permutational multivariate analysis of variance (PERMANOVA) are shown in Additional file 3.

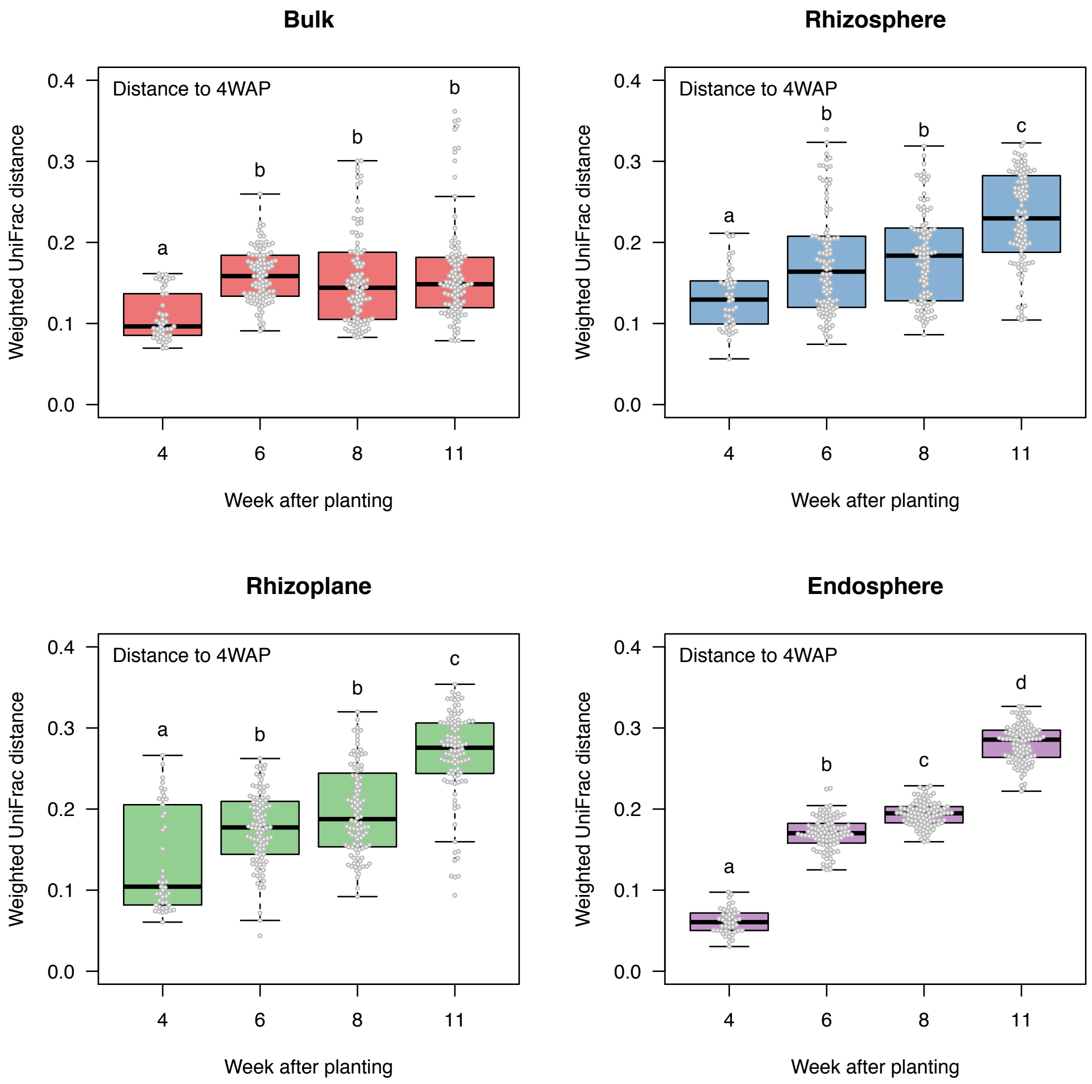

Supplementary Figure S8. Time-course shifts of dissimilarity of bacterial communities during plant development.

Different letters above boxes indicate significant differences among different growth stages ($p < 0.05$, Wilcoxon rank-sum test with the Bonferroni correction).

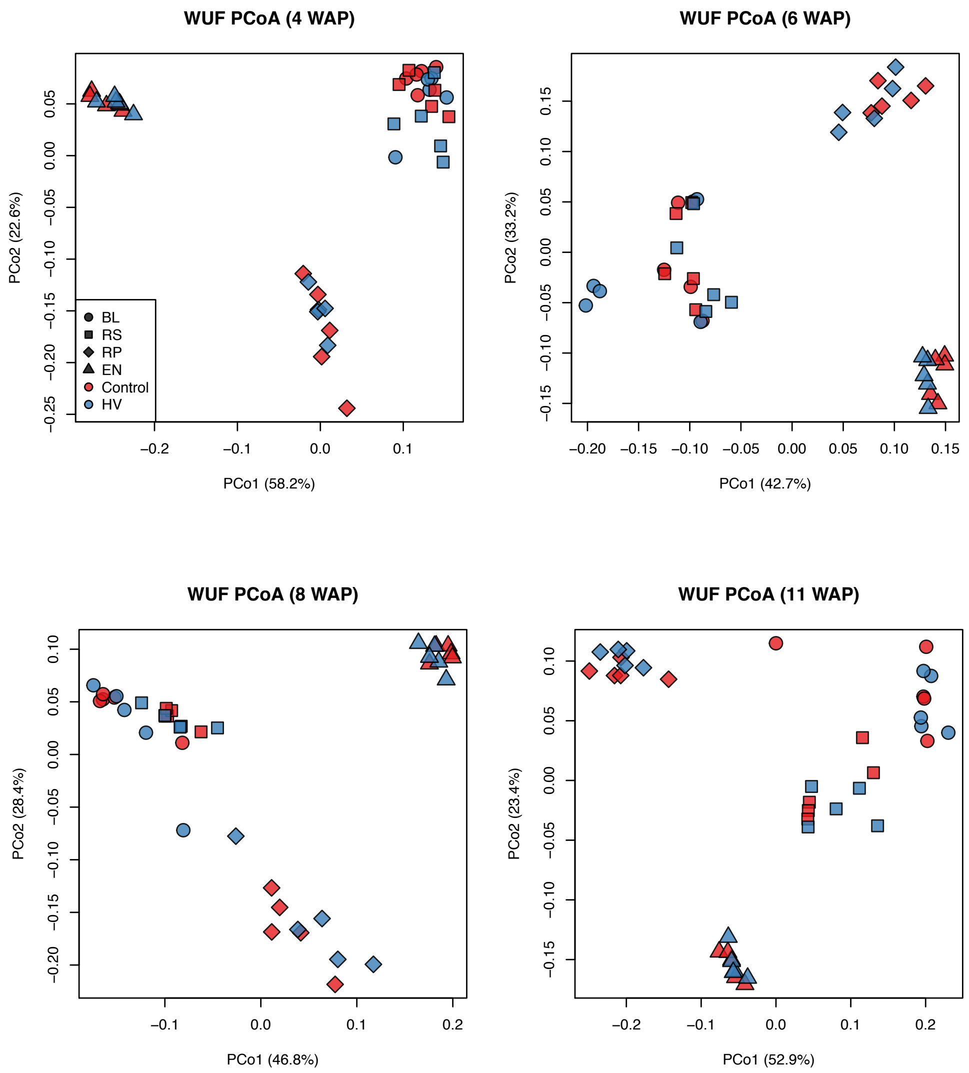

Supplementary Figure S9. Dissimilarity of bacterial communities between the control and HV plot.

The principal coordinate analysis (PCoA) of weighted UniFrac distances were performed. Results of the permutational multivariate analysis of variance (PERMANOVA) are shown in Additional file 3. BL, bulk soil; RS, rhizosphere; RP, rhizoplane; EN, endosphere.

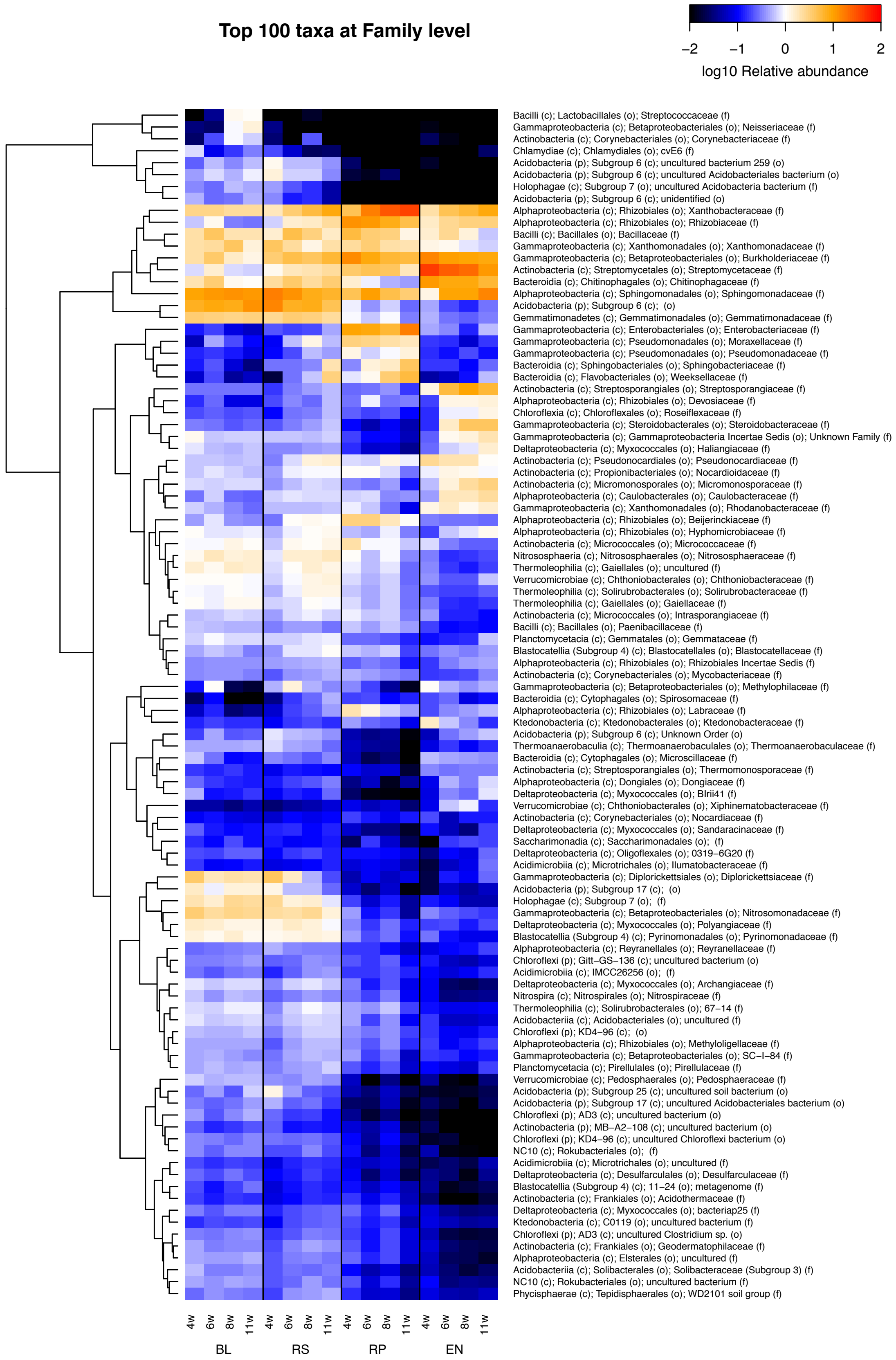

Supplementary Figure S10. Distribution of top 100 taxa at family levels in the root-associated environments.

BL, bulk soil; RS, rhizosphere; RP, rhizoplane; EN, endosphere.

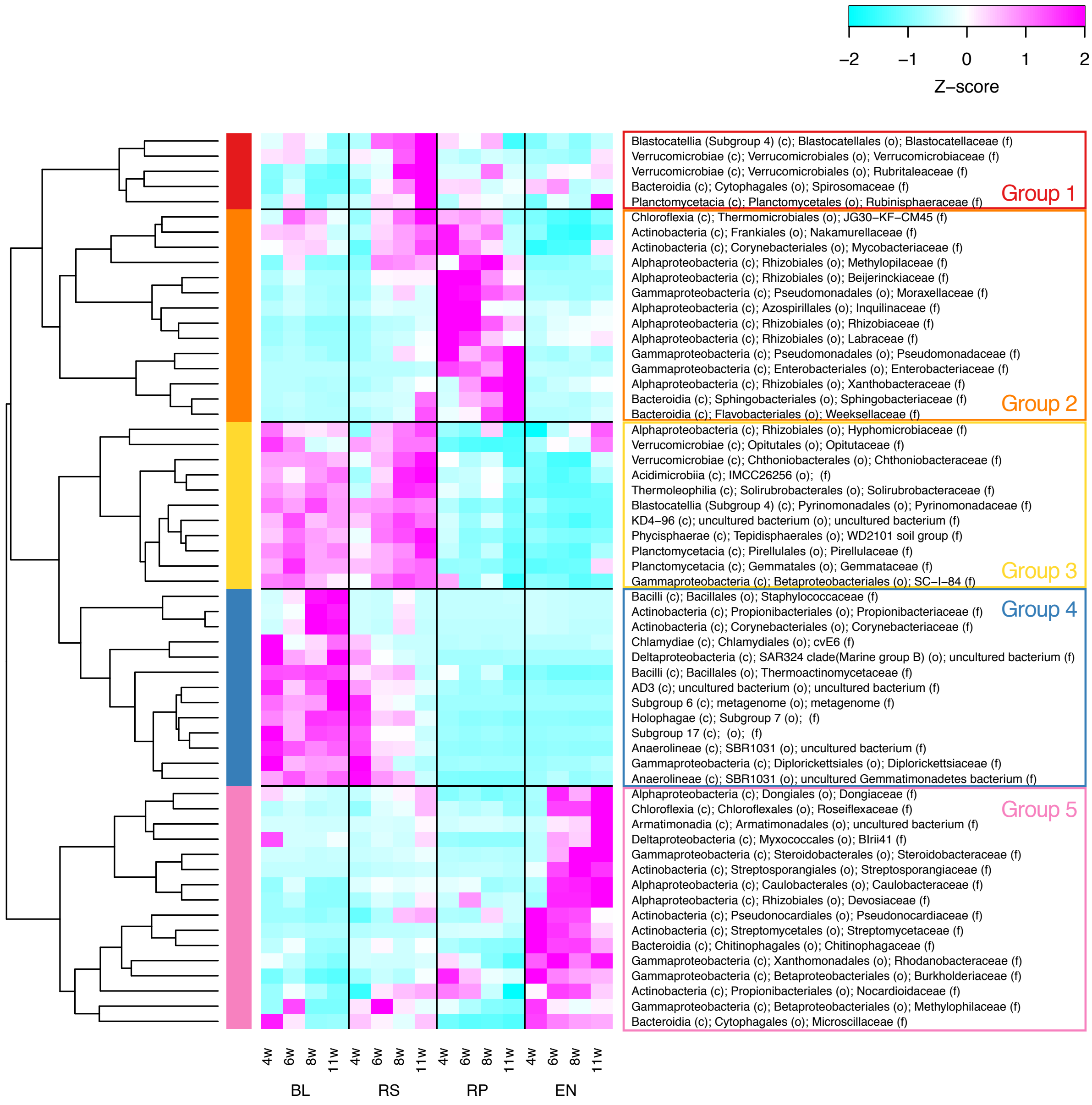

Supplementary Figure S11. Distribution of differentially abundant taxa between the bulk soil and rhizosphere. Differentially abundant 59 taxa between the bulk soil and rhizosphere at 11 weeks after planting (FDR < 0.01, ALDEx2). Values are expressed as the scaled values of relative abundance of each taxon. They are classified into five groups based on the distribution pattern.

Group 1: Taxa enriched in the rhizosphere.

Group 2: Taxa enriched in the rhizosphere and rhizoplane.

Group 3: Taxa enriched in the rhizosphere and depleted in the rhizoplane and endosphere.

Group 4: Taxa depleted in the rhizosphere.

Group 5: Taxa enriched in the rhizosphere and endosphere.

BL, bulk soil; RS, rhizosphere; RP, rhizoplane; EN, endosphere.

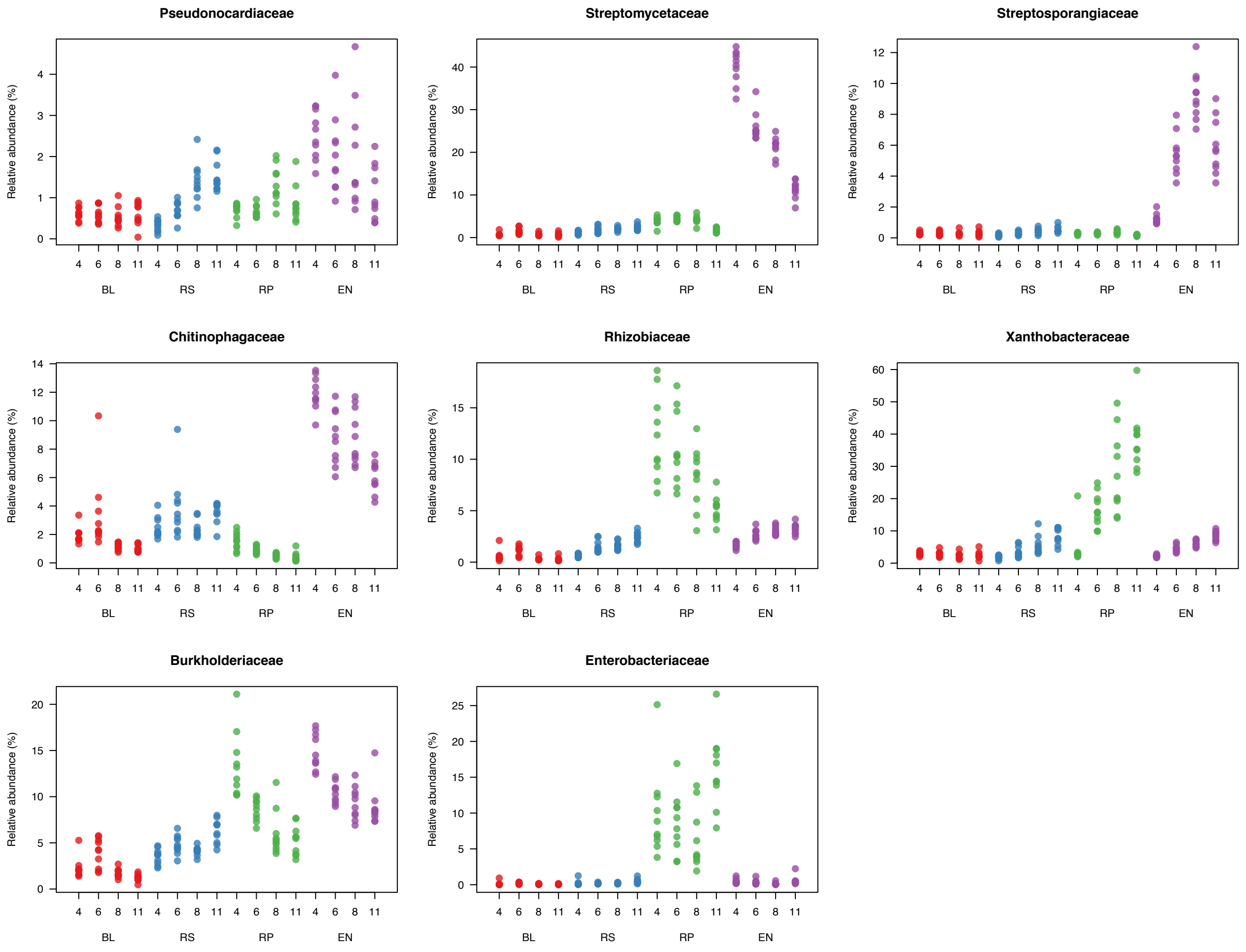

Supplementary Figure S12. Distribution of differentially abundant dominant taxa.

Of top 15 dominant taxa in root-associated compartment, 8 taxa were significantly more abundant in the rhizosphere than in the bulk soil (FDR < 0.01, ALDEx2). BL, bulk soil; RS, rhizosphere; RP, rhizoplane; EN, endosphere.

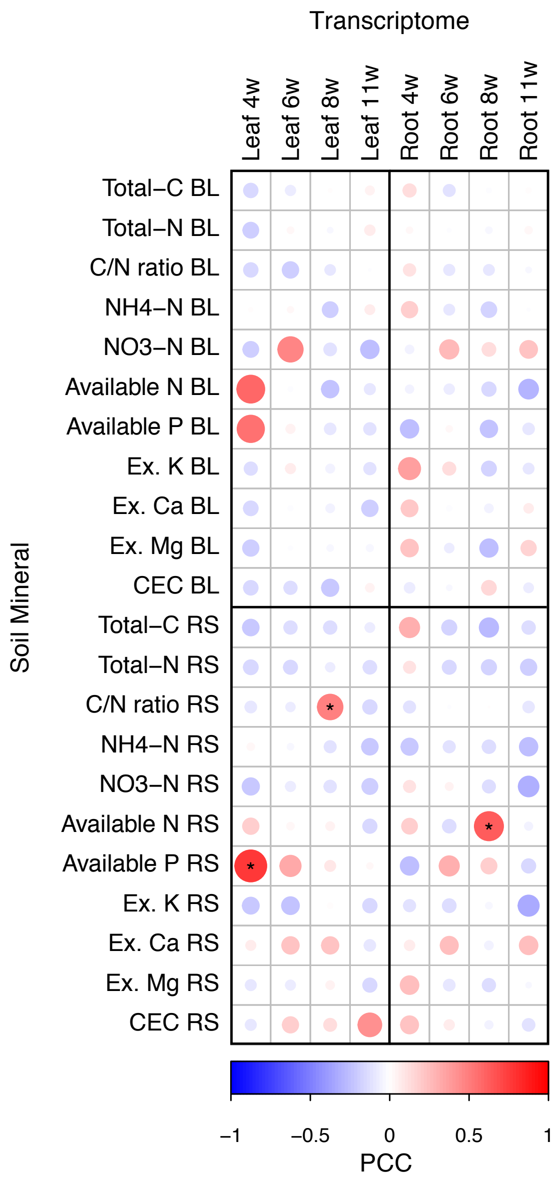

Supplementary Figure S13. Mantel's statistics between the plant transcriptome and the soil environment.

Mantel's test was performed using the correlation distance matrix of gene expression and the Euclidian distance matrix of each mineral content at each sampling time. Asterisks (*) show significant correlations (p < 0.01). PCC, Pearson correlation coefficient; BL, bulk soil; RS, rhizosphere; Ex, exchangeable; CEC, cation exchange capacity.

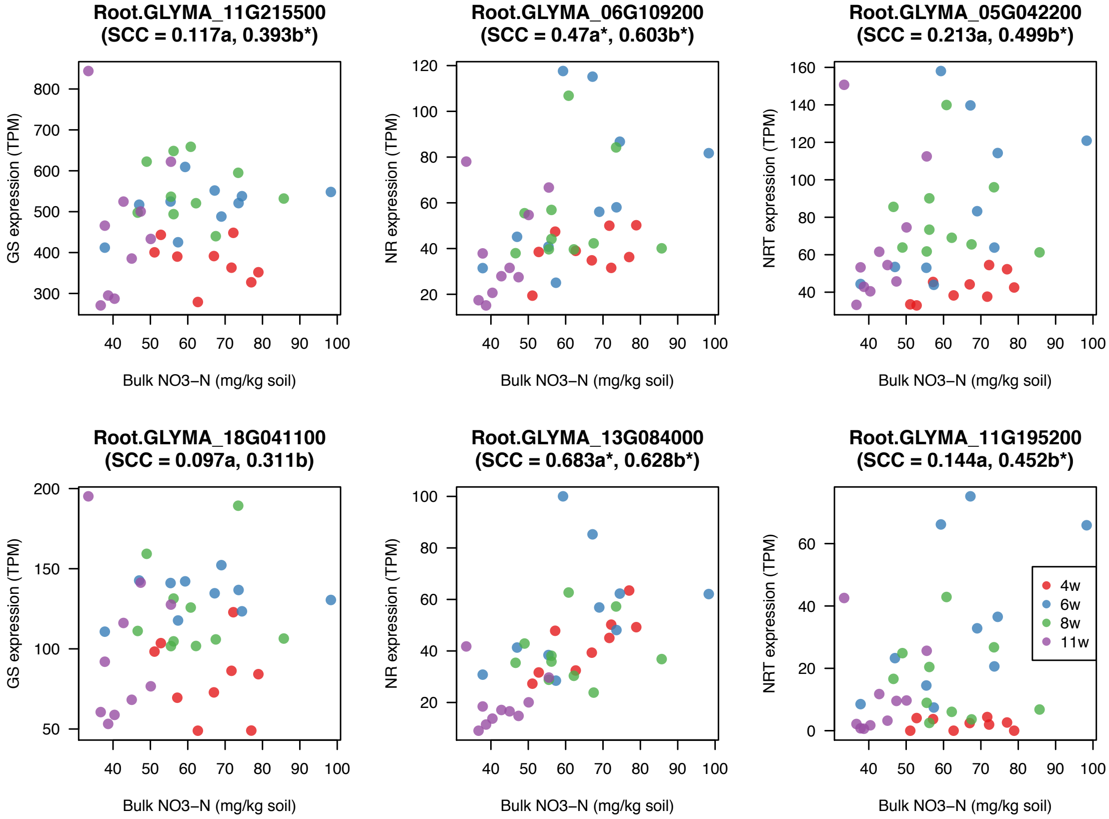

Supplementary Figure S14. Correlation between the expression of nitrogen responsive genes and the content of NO_3-N in the bulk soil.

Scatter plots of gene expression levels and NO_3-N contents, and Spearman correlation coefficients (SCC). SCC values with “a” indicate correlation during 4–11 WAP and those with “b” during 6–11 WAP. Asterisks (*) show significant correlations ($p < 0.05$).

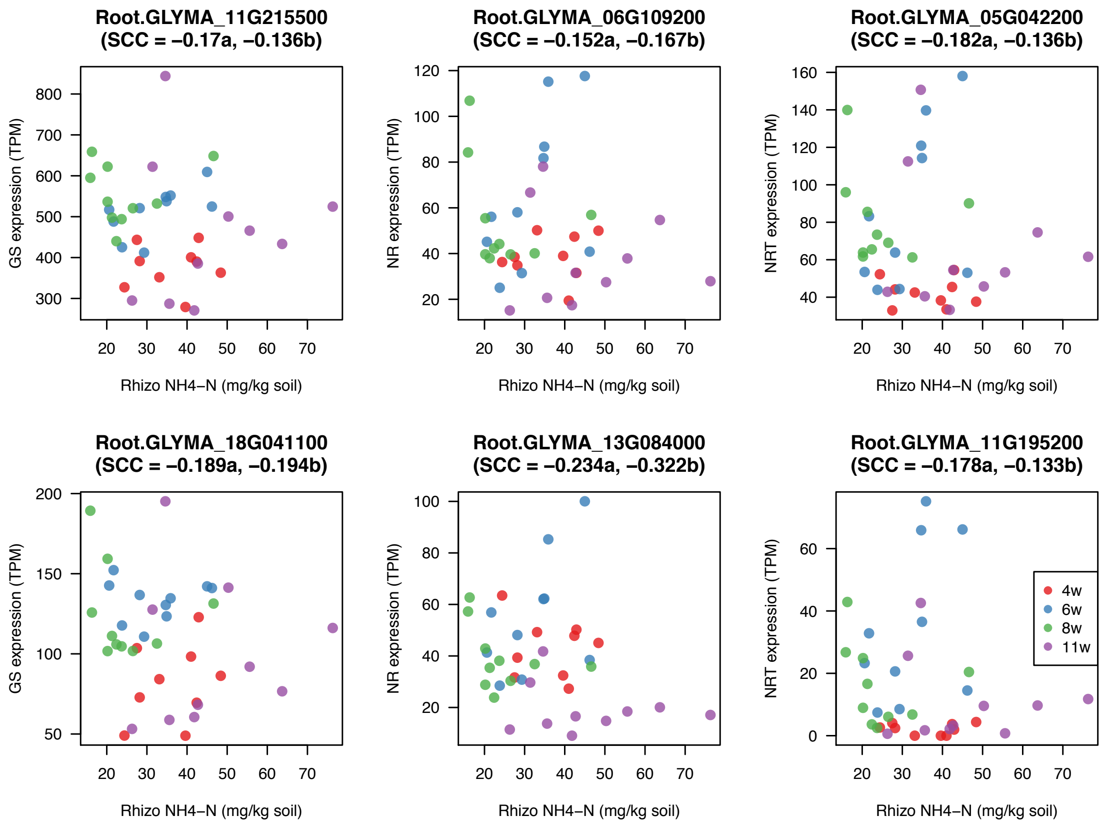

Supplementary Figure S15. Correlation between the expression of nitrogen responsive genes and the content of NH_4-N in the rhizosphere soil.

Scatter plots of gene expression levels and NH_4-N contents, and Spearman correlation coefficients (SCC). SCC values with “a” indicate correlation during 4–11 WAP and those with “b” during 6–11 WAP. A significant correlation was not detected ($p < 0.05$).

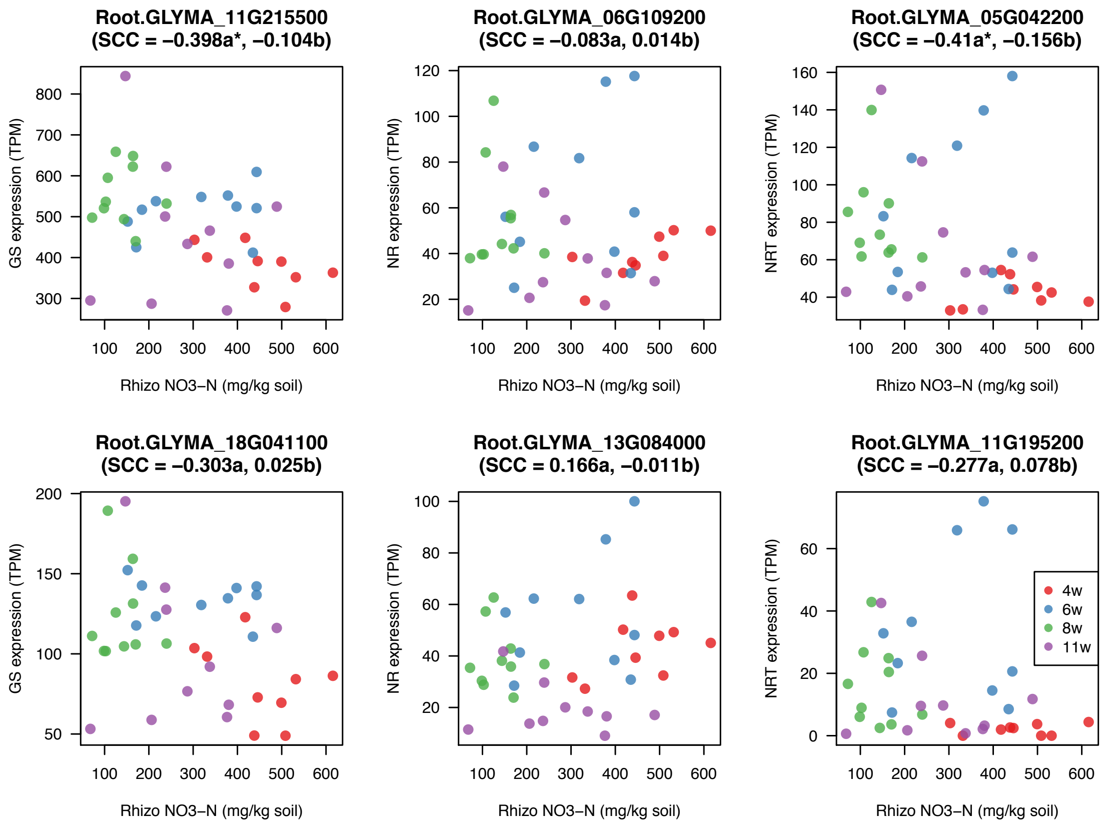

Supplementary Figure S16. Correlation between the expression of nitrogen responsive genes and the content of NO_3-N in the rhizosphere soil.

Scatter plots of gene expression levels and NO_3-N contents, and Spearman correlation coefficients (SCC). SCC values with “a” indicate correlation during 4–11 WAP and those with “b” during 6–11 WAP. Asterisks (*) show significant correlations ($p < 0.05$).

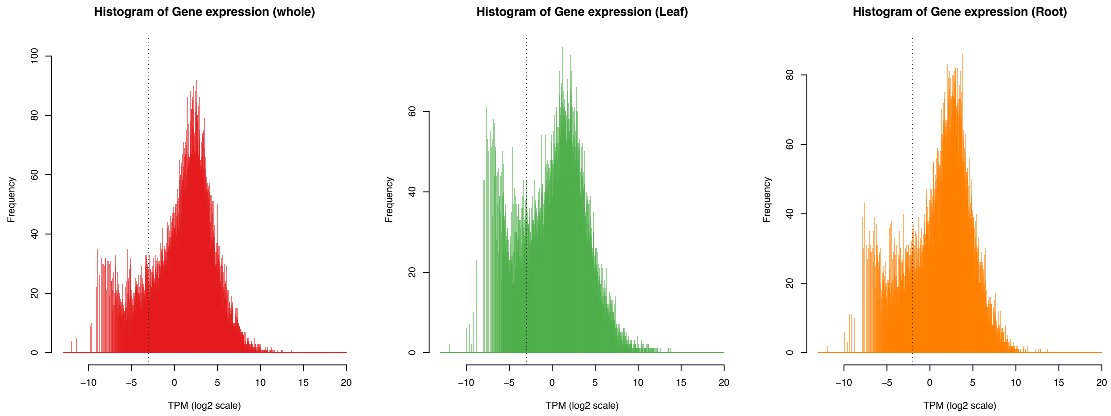

Supplementary Figure S17. Distribution of the means of gene expression levels.

Mean expression levels were expressed in the log2 scale.
